# Copper oxide nanostructured thin films processed by SILAR for optoelectronic applications

**DOI:** 10.1039/d2ra06303d

**Published:** 2022-11-16

**Authors:** Md Abdul Majed Patwary, Md Alauddin Hossain, Bijoy Chandra Ghos, Joy Chakrabarty, Syed Ragibul Haque, Sharmin Akther Rupa, Jamal Uddin, Tooru Tanaka

**Affiliations:** Department of Chemistry, Physical Chemistry Research Laboratory, Comilla University Cumilla 3506 Bangladesh mamajedp@gmail.com; Department of Electrical and Electronic Engineering, Saga University Saga 840-8502 Japan; Department of Physics, Comilla University Cumilla 3506 Bangladesh; Center for Nanotechnology, Department of Natural Sciences, Coppin State University Baltimore MD USA

## Abstract

The lack of high-functioning p-type semiconductor oxide material is one of the critical challenges that face the widespread performance of transparent and flexible electronics. Cu_*x*_O nanostructured thin films are potentially appealing materials for such applications because of their innate p-type semi-conductivity, transparency, non-toxicity, abundant availability, and low-cost fabrication. This review summarizes current research on Cu_*x*_O nanostructured thin films deposited by the SILAR technique. After a brief introduction to the advantages of Cu_*x*_O semiconductor material, diverse approaches for depositing and growing such thin films are discussed. SILAR is one of the simplest deposition techniques in terms of better flexibility of the substrate choice, the capability of large-area fabrication, budget-friendly, deposition of stable and adherent film, low processing temperature for the film fabrication as well as reproducibility. In addition, various fabrication parameters such as types of copper salts, pH of precursors, number of cycles during immersion, annealing of as-deposited films, doping by diverse dopants, and growth temperature affect the rate of fabrication with the structural, electrical, and optical properties of Cu_*x*_O nanostructured thin films, which led the technique unique to study extensively. This review will include the recent progress that has recently been made in different aspects of Cu_*x*_O processed by the SILAR. It will describe the theory, mechanism, and factors affecting SILAR-deposited Cu_*x*_O. Finally, conclusions and perspectives concerning the use of Cu_*x*_O materials in optoelectronic devices will be visualized.

## Abstract 2

Cu_*x*_O nanostructured thin films are potentially appealing materials for many applications. The deposition technique, SILAR, explored in this paper offers many advantages.

## Graphical abstract

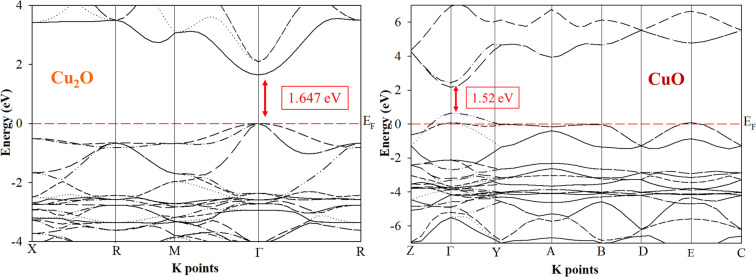

## 1. Introduction

### 1.1 Background

Copper (Cu) and copper oxide (Cu_*x*_O) thin films have been studied extensively due to their potential application in semiconductor technology long before the Ge and Si era started, and researchers have faced much more difficult to work with this oldest material ever. The n-type window layer semiconductors such as ZnO, ITO, FTO, and GaN with large bandgap energies have already achieved outstanding optical as well as electronic transport properties. Consequently, the effort of detecting new, prospective p-type absorber layers for optoelectronics devices has led to intensive research.

Cu_*x*_O semiconductors are very attractive and have been broadly studied in both theoretical analysis and investigations into applied executions of nano or optoelectronic devices due to their chemically stable nature, nontoxicity, relative abundance, potential particle size effects, excellent performance as a catalyst, and fulfill all the requirements for low-cost manufacturing at ambient conditions, which have high potential usage in energy storage, conversion, and next-generation rechargeable lithium-ion batteries.^1–6^ Furthermore, Cu_*x*_O nanostructures are extensively used in other diverse applications, including photovoltaics,^7^ photodetectors,^8^ nanofluid,^9,10^ energetic materials,^11^ field emissions,^12^ supercapacitors,^13,14^ biosensors,^15,16^ gas sensors,^17,18^ photocatalysis,^19,20^ removal of inorganic pollutants,^21,22^ and magnetic storage media.^23,24^

Both the Cu_2_O and CuO show direct transition nature with a direct band gap of around 2.1 and 1.5 respectively, having a high extension coefficient of above 10^5^ cm^−1^. Since the theoretical limit of the energy conversion efficiency of Cu_2_O and CuO is as high as 20 and 29%, respectively under air mass (AM) 1.5 solar illumination, numerous efforts were done to increase the efficiency of Cu_*x*_O solar cells, but the performance remains very poor.^25^ In the case of Cu_2_O solar cells, it is not more than 8.1%,^7^ whereas in the case of CuO it is lower and still about 3%.^26^ Toward the large area fabrication, it is crucial to establish the thin film growth technique for Cu_*x*_O. Thus, the research of Cu_*x*_O thin films has both high-tech and scientific consequences.

Cu_*x*_O nanostructured thin films have been synthesized by various approaches like electrodeposition,^27^ electron beam evaporation,^28^ magnetron sputtering,^29–31^ molecular beam epitaxy,^32^ sol–gel,^33^ solution growth,^34^ spin coating,^35^ successive ionic layer adsorption and reaction (SILAR),^36,37^ thermal evaporation,^38^ and vapor deposition.^39^ Among all the deposition methods, SILAR is one of the simplest methods in terms of better flexibility on substrate choice, the capability of large area fabrication and deposition of the stable and adherent film, low processing temperature for film fabrication as well as reproducibility.^40^ This technique is very budget friendly since it does not require any sophisticated equipment. Moreover, various fabrication parameters such as pH, annealing temperature and time, doping elements, the concentration of precursor solutions, and temperature of the precursor solutions affect the rate of fabrication as well as the structural, optical, and electrical properties of the fabricated thin films led the technique unique to study in an extensive manner.

More than a few reviews of different aspects of Cu_*x*_O-based optoelectronics have been published based on the fabrication technique but still no such report for the SILAR technique. This paper concerns the progress that has recently been made in diverse aspects of Cu_*x*_O-based thin films processed by the SILAR method, following the introduction in section one, several deposition techniques are reviewed in section two. The third section of this paper describes the theory and mechanism of Cu_*x*_O-based thin films fabricated by the SILAR method. The fourth section, which incorporated the core focus of this review, leads to the factors that affect SILAR-based Cu_*x*_O deposition which is followed by the application of Cu_*x*_O in section five. Finally, conclusions and perspectives concerning the use of Cu_*x*_O in optoelectronic devices are presented.

### 1.2 Properties of copper oxides (Cu_*x*_O)

Cu_2_O exists as a simple cubic Bravais lattice^8–10^ with a space group of (*Pn*3*m*) or (*O*4_h_). Each unit cell consists of six atoms, the four Cu atoms are in a face-centered cubic lattice while the two O atoms are at the tetrahedral positions creating a body-centered cubic sublattice. Thus, O atoms are fourfold coordinated with Cu atoms as closest neighbors, and Cu atoms are linearly coordinated with two O atoms as closest neighbors as shown in Table 1. On the other hand, the unit cell of CuO fits into a monoclinic structure with the space group *C*2/*c* and the lattice parameters are represented in the table (PDF No. 89-5898). In each CuO unit, there exist four Cu–O bonds. As demonstrated in the table, in a unit, each Cu atom is surrounded by the four closest coplanar O atoms. The four O atoms are positioned at the angles of an almost rectangular parallelogram, which then unites another two O atoms to shape a highly distorted octahedron. The O atom is enclosed by the four closest Cu atoms positioned at the angle of a tetrahedron.

**Table 1:** Crystallographic properties of Cu_2_O and CuO^41^

Parameters		Cu_2_O	CuO
Structure: Cu(i)-yellowish, Cu(ii)-greyish black, O-red		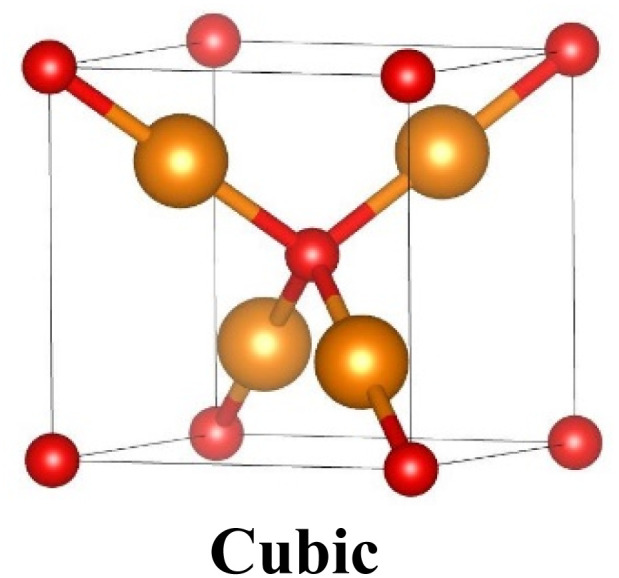	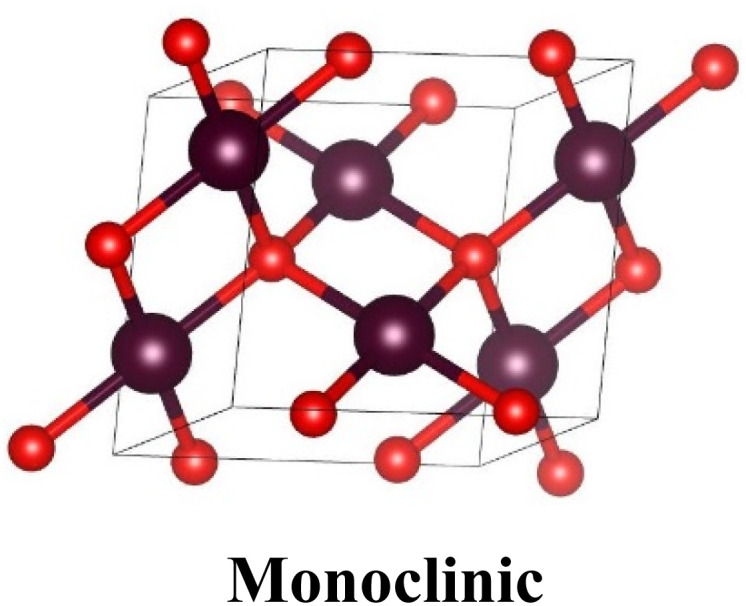
Unit cell		*a* = *b* = *c* = 4.26 Å	*a* = 4.6837 Å, *b* = 3.4226 Å, *c* = 5.1288 Å
		*α* = *β* = *γ* = 90°	*α*, *γ* = 90°, *β* = 99.54°
Space group		*Pn*3̄*m* (224)	*C*2/*c* (15)
Bond length, Å	Cu–O	1.849	1.96
	O–O	3.68	2.62
	Cu–Cu	3.012	2.90
Cell volume, Å^3^		77.83	81.08
Formula weight		143.14	79.57
Density, g cm^−3^		5.749–6.140	6.515
Melting point, °C		1235	1201

### 1.3 Band-structure calculation

**Fig. 1:**
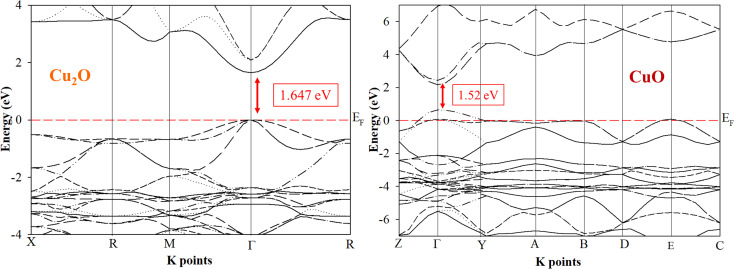
Band structures of Cu_2_O and CuO unit cells are drawn by CASTEP using LDA + *U*.

**Fig. 2:**
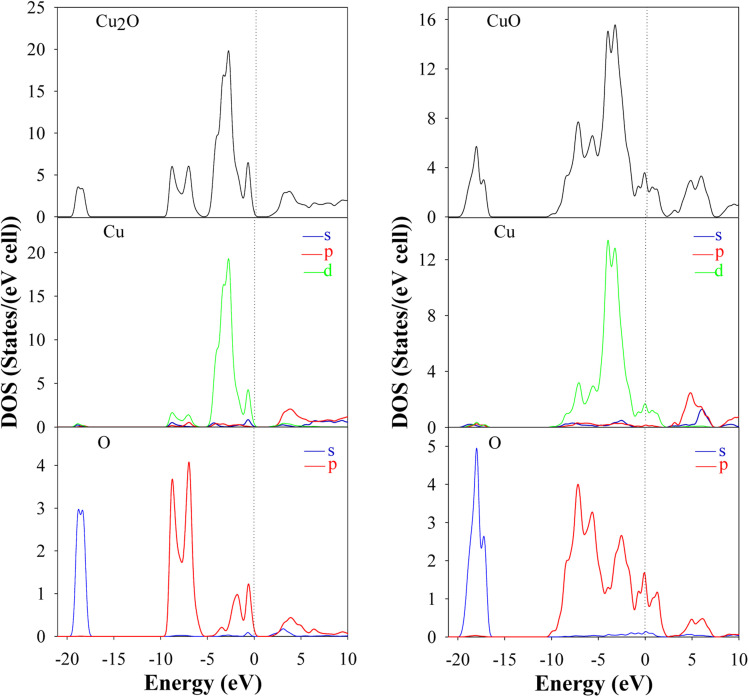
DOS and PDOS of Cu_2_O and CuO unit cells are drawn by CASTEP using LDA + *U*.

Ab initio calculations are mandatory to understand the optical and electronic properties of the Cu_*x*_O systems. But there is a challenge for standard *ab initio* investigations based on DFT for both Cu_2_O and CuO. The exchange–correlation function is the crucial ingredient in the theoretical description. Fig. 1 and 2 represent the band structures, density of states (DOS), and partial density of states (PDOS) of the Cu_2_O and CuO compounds. The results were simulated for both Cu_2_O and CuO unit cells using CASTEP software within the LDA + *U* and the calculated bandgaps were found as 1.647 and 1.52 eV respectively.^42^

## 2. Thin film deposition process

### 2.1 Physical deposition methods

The physical deposition methods have diverse techniques to attain thin films with good quality. It can be summarized with the raw materials, deposition conditions as well as cost of production as shown in Table 2.

**Table 2:** Physical deposition techniques with the requirements during deposition

Physical deposition						
Techniques	Raw materials	Conditions	Substrate	Film quality	Budget	Ref.
Thermal evaporation	Red Cu_2_O powder	Base pressure: 5 × 10^−4^ Pa	Glass tantalum SiO_2_	—	High	43
		Temperature: 300 °C				
		Annealing temperature: 500 °C				
Electron beam evaporation	Cu_2_O pellets	Deposition time: 5–16 min	Glass	Moderate	High	44
		Substrate temperature: 200 °C				
		Accelerating voltage: 2, 4 and 6 kV				
		Evaporation pressure: 3 × 10^−2^ Pa				
		Ultimate pressure: 4 × 10^−4^ Pa				
		Filament current: 30 mA				
Pulsed laser deposition	Cu, O_2_	Substrate temperature: 25–400 °C	Quartz ITO NaCl	Excellent	High	45
		*p*_O_2__: 0–10 mTorr				
		Vacuum chamber pressure: Torr				
Molecular beam epitaxy	Cu, O_2_	Incident O^+^ beam energy: 50 eV	MgO	Excellent	High	46
		Substrate temperature: 100–400 °C				
		Cu flux: 2.5 × 10^13^ to 1.6 × 10^14^ atoms per cm^2^ s				
		Base pressure: 3 × 10^−10^ torr				
		Total pressure: 3 × 10^−9^ to 2 × 10^−8^				
		O^+^ flux: 2.7 × 10^14^ atoms per cm^2^ s				
Ion plating evaporation	Cu, O_2_	Base pressure: 10^−4^ Pa	Glass	Excellent	High	47
		O_2_ flow rate: 15–45 sccm				
		N_2_ flow rate: 0–6 sccm				
		RF power: 300 W				
		Annealing temperature: 300 °C				
		Substrate temperature: 25 °C				
Direct current (DC) sputtering	Cu, O_2_	Deposition pressure: 6.3 × 10^−3^ torr	Glass	Excellent	High	48 and 49
		Base pressure: 6 × 10^−6^ torr				
		Sputtering power: 60 W				
		Ar gas pressure: 20 sccm				
		*p*_O_2__: 8.0 × 10^−4^ to 1.8 × 10^−3^ torr				
	Cu, O_2_	Sputtering power: 10–40 W	Glass stainless steel			
		Ar flow rate: 15 sccm				
		O_2_ flow rate: 10 sccm				
		Substrate temperature: 300 °C				
		Ambient gas pressure: 0.045 Pa				
Radio frequency (RF) sputtering	Cu, O_2_	Substrate temperature: 300 °C	Glass	Excellent	High	50
		Ar flow rate: 10 sccm				
		O_2_ flow rate: 0–2 sccm				
		Deposition time: 60 min				
		Background pressure: <3 × 10^−4^ Pa				
		Working pressure: 1.7–1.8 Pa				

### 2.2 Chemical deposition methods

Likewise, diverse chemical deposition techniques with the deposition condition, raw materials, cost of production, the usual use of substrate *etc.* are discussed in Table 3 as shown below:

**Table 3:** Chemical deposition techniques with the requirements during deposition

Techniques	Raw materials	Chemical deposition	Substrate	Film quality	Budget	Ref.
		Conditions				
Sol–gel	CuCl_2_·2H_2_O, CH_3_OH, (CH_2_CH_2_OH)NH, glucopone, ethylene glycol	Rotating speed: 2000 rpm	TiO_2_	Good	Low	51 and 52
		Annealing temperature: 200–400 °C				
	Cu(ii) acetate, H_2_N(CH_2_)_2_OH, 2-methoxyethanol, poly (ethylene glycol)	Rotating speed: 1000 and 1500 rpm	FTO Si			
		Annealing temperature: 350, 500 °C				
Chemical bath deposition	Cu(NO_3_)_2_·3H_2_O, triethanolamine, hydrazine hydrate	Temperature: 30 °C	Glass	Excellent	Low	53 and 54
	Cu, HNO_3_, HF, C_2_H_5_OH, Na_2_SO_4,_ CH_3_COCH_3_	Temperature: 30 °C	Cu			
		Molar ratio: HNO_3_ : HF-10 : 1 to 135 : 1				
		HNO_3_ conc: 0–1.2 mmol L^−1^				
		Water bath time: 2–6 days				
		Water-bath temperature: 10–45 °C				
SILAR	CuSO_4_·5H_2_O, NaOH, Na_2_S_2_O_3_·5H_2_O	Temperature: 70 °C	Glass FTO	Excellent	Low	36 and 37
		pH: 2.35–7.33				
	CuSO_4_·5H_2_O, NaOH, Na_2_S_2_O_3_·5H_2_O	Temperature: 70 °C	Glass FTO			
		pH: 4.50–7.95				
		No. of cycles: 20–80				
		Annealing temperature: 75–350 °C				
Spray pyrolysis	Cu(CH_3_COO)_2_·H_2_O, (CH_3_)_2_CHOH, C_6_H_12_O_6_	Temperature: 200–350 °C	Glass	Excellent	Low	55 and 56
		Deposition time: 45 min				
		No. of cycles: 450				
	Cu(CH_3_COO)_2_·H_2_O, C_6_H_12_O_6_, (CH_3_)_2_CHOH	Temperature: 200–350 °C	Glass			
		Glucose conc: 0–0.08 M				
		Isopropanol volume (%): 0–100				
		Cu salt conc: 0–0.08 M				
Electrodeposition	CuSO_4_·5H_2_O, lactic acid, NaOH	Temperature: 30 and 60 °C	Ti	Excellent	Low	57–60
		pH: 9, 12				
		Applied potential: −150 to −800 mV				
	CuSO_4_·5H_2_O, tri-sodium citrate dehydrate, C_6_H_5_Na_3_O_7_; KOH	pH: 11	FTO			
		Time: 20–60 min				
		Applied potential: −0.4 V				
		Temperature: 30 °C				
	Cu(CH_3_COO)_2_·H_2_O, CH_3_COONa·3H_2_O	Temperature: 20–80 °C	ITO			
		Time: 2–80 min				
		NaCl conc: 1–10 mM				
		Potential: −0.1 to −0.4 V				
	Cupric acetate, sodium acetate, CH_3_COOH, NaOH	Temperature: 20, 55 °C	Ti ITO Pt			
		Deposition time: 45 min				
		pH: 5.4–7.49				
		Applied potential: −200 to −400 mV				
		Cu(ii) acetate con.: 0–16 mM				
Chemical vapor deposition (CVD)	Cu dipivaloylmethanate, Cu(C_11_H_9_O_2_)_2_; O_2_	O_2_ flow rate: 1 and 300 cm^3^ min^−1^	Borosilicate glass	Excellent	High	61–63
		*p*_O_2__: 1.689 × 10^2^ and 5.07 × 10^4^ Pa				
		Temperature: 300 and 500 °C				
	*N*,*N*′-di-*sec*-butylacetamidinato)Cu(i), Cu-(*sec*-Bu-Me-amd)]_2_; DI H_2_O	Substrate temperature: 125–225 °C	Si waferGlassy carbon SiO_2_ glass			
		Process pressure: 1–10 torr				
		Vapor flow rate: 5 sccm				
		N_2_ flow rate: 100 sccm				
	Cu(ii) acetylacetonate, Cu(C_5_H_7_O_2_)_2_; O_2_	Pressure: 5–200 sccm	Sapphire MgO			
		Temperature: 350–500 °C				
Atomic layer deposition (ALD)	Cu(CH_3_COO)_2_·H_2_O, Cu(CH_3_COO)_2_, H_2_O vapor	Temperature: 180–220 °C	Glass Si	Excellent	High	64 and 65
		Rector pressure: 10 mbar				
		N_2_, H_2_O and O_2_ flow rate: 400 sccm				
		Deposition cycles: 500–7000				
	Cu(ii)-bis-(dimethylamino-2-propoxide), O_3_	*p*_O_2__: 34 Pa	SiO_2_/Si			
		Substrate temperature: 112–165 °C				
		Deposition cycles: 500–10000				

### 2.3 Advantages and disadvantages of deposition techniques

Till now, a lot of deposition techniques are available to fabricate high-quality thin films having diverse applications. For a better understanding, the advantages, and disadvantages of some of the chemical deposition techniques such as chemical bath deposition (CBD), atomic layer deposition (ALD) as well as spin coating are summarized to understand the potentiality of the SILAR method in Table 4.

**Table 4:** Involved advantages and disadvantages of the deposition techniques

Techniques	Merits	Demerits
CBD	Simple and cost effective	Precipitation occurs in the bath causing serious problems
	Stoichiometric deposition^66^	Materials are lost^70^
	Low fabrication temperature	Films are badly cohered onto the substrate
	Capability of depositing large area films (∼10 cm^2^)^67^	Produces powdery films
	Various types of substrates are used^68^	Failed to control film thickness
	Tuning film qualities by controlling growth parameters	Deposited films are contaminated though organic additives
	Deposition of ternary and quaternary compounds^69^	Opposite ions present in the reaction bath
SILAR	Facile and economical	Perfect adsorption of ions requires on the substrate surface
	Potentiality to grow large-surface films (∼10 cm^2^)^71^	Substrate surface must be balanced completely through precursor solution^73^
	Reproducibility	
	Any kind of substrate can be used	
	No need to use sophisticated instrument or vacuum pump	
	No precipitation occurs in the bath	
	Synthesis of doped, ternary, and quaternary compounds	
	Does not need premium quality target	
	Controlling on film thickness	
	Avoid unnecessary heating	
	Minimization of dislocation density by controlling deposition parameters^72^	
	Fabricated stable and sticky films^36^	
ALD	Films thicknesses are under controlled	Sluggish deposition process
	Layer by layer film deposition	Highly refined substrate is required
	Deposition can be performed at relatively low temperature	Instrument and substrate are highly priced
	Soft substrates can be used	Several trials required to set optimize film growth condition
	Ability to use thermally unstable precursors due to slow degradation^74,75^	Process is restricted to non-volatile compounds
		Unfavorable for heat sensitive biological substrates^76,77^
Spin coating	Dominated over film thicknesses	Hindered to use multiple substrates at a time
	Easy and effortless	Restricted to utilize big substrate
	Affordable	Low material productivity
	Fabricated stable cohered films	Inexpensive with respect to photoresist and substrate size
	Deficit of coupled variables	95–98% materials are wasted^80^
	Reproducibility^78,79^	Less effective in nanotechnology due to quick drying^81^
Solvothermal	Dominate over crystallinity of the deposited films	Requirement of expensive autoclave
	Produces intermediate to specific quality films	Safety issues
	Potentiality to synthesize solid-state materials	Impossible to observe *in situ* reaction process^82^
		Less dominance over particle size^83^

## 3. Theory and mechanism of SILAR process

SILAR is an extensively applied technique to fabricate high-quality metal oxide or halide thin films.^84,85^ During deposition, successive ionic layer adsorption and reaction of the ions take place at the solid–solution interface of the substrate. Thus, the thin film of the compound, A_*x*_B_*y*_ is deposited onto the substrate surface by dint of the adsorbed cations, *x*A^*p*+^ and anions, *y*B^*q*−^ due to the following heterogeneous chemical reaction:*x*[A(L_*n*_)]^*p*+^ + *p*P^*x*−^ + *q*Q^*y*+^ + *y*B^*q*−^ → A_*x*_B_*y*_ + *q*Q^*y*+^ + *p*P^*x*−^where, *x*, *p*, *q*, *y* and *p*^+^, *x*^−^, *y*^+^, *q*^−^ are the number and charges of the corresponding ions A (metal ions), P (cationic precursor), Q (anionic precursor) and B (anions) respectively.^85,86^ Sometimes, the ligands, L_*n*_ are a necessity to complete the reaction.^87–90^

In the case of Cu_*x*_O film deposition mechanism, salts of Cu^2+^ are used to deposit copper oxide thin films. In most of the research on Cu_2_O, firstly copper(i) thiosulfate complex is formed by the redox reaction between Cu^2+^ and S_2_O_3_^2−^ ions which results in a colorless solution. The corresponding reactions are:

Oxidation half-reaction:2S_2_O_3_^2−^ → S_4_O_6_^2−^ + 2e^−^

Reduction half-reaction:2Cu^2+^ + 2e^−^ → 2Cu^+^

Overall reaction:2Cu^2+^ + 4S_2_O_3_^2−^ → 2[Cu(S_2_O_3_)]^−^ + S_4_O_6_^2−^

**Chart 1:**
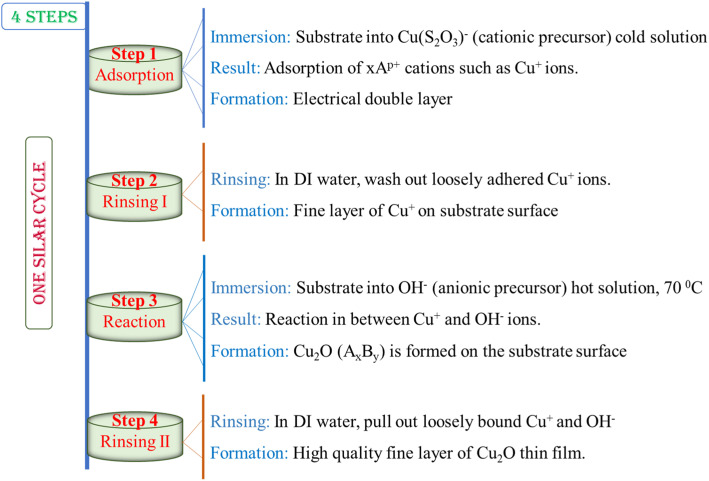
Representation of different steps during a SILAR cycle.

In the above reactions, [Cu(S_2_O_3_)]^−^ the complex solution is regarded as the cationic precursor solution (cold solution) while NaOH is the anionic precursor solution, which is being kept at 70 °C (hot solution).^91^ When the substrate is immersed in the hot solution, OH^−^ ions are adsorbed onto the substrate and subsequently dipping into the cold solution results in the adsorption of Cu^+^ ions. Thus, one SILAR cycle is completed and Cu_2_O thin film is formed due to the reaction between Cu^+^ and OH^−^ ions. Rinsing is carried out after every immersion to exclude loosely adhered particles. The number of cycles as well as dipping time varies based on required film thicknesses. Corresponding reactions are given below ^92^ and the growth mechanism is schematically represented in Chart 1.[Cu(S_2_O_3_)]^−^ → Cu^+^ + 2S_2_O_3_^2−^2Cu^+^ + 2OH^−^ → Cu_2_O(s) + H_2_O

**Chart 2:**
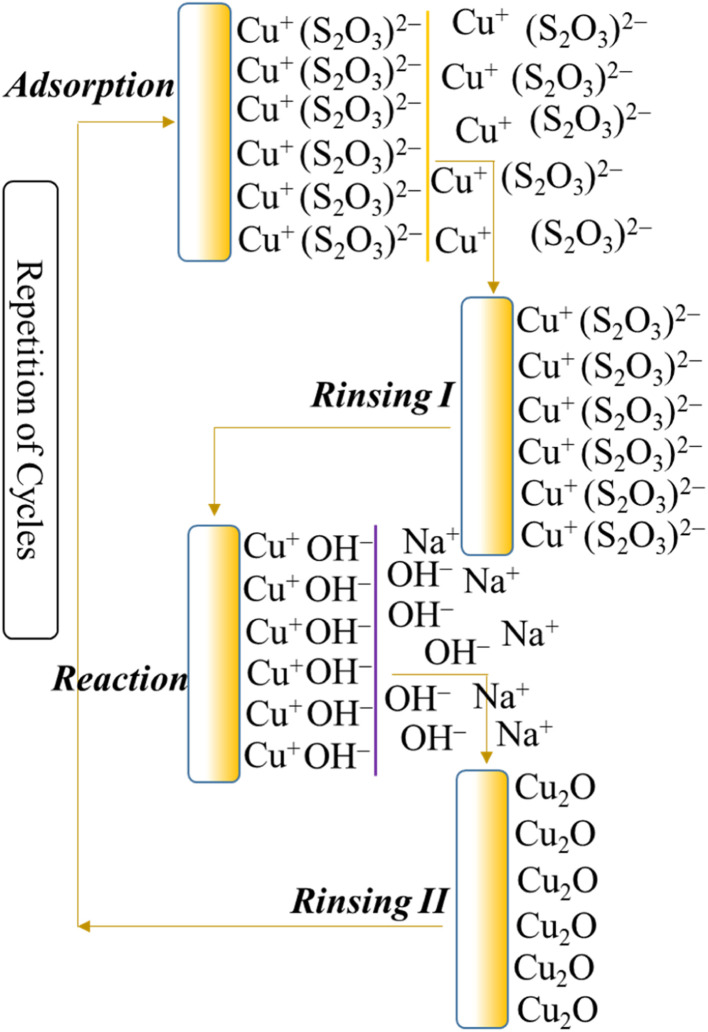
Schematic presentation of the deposited Cu_2_O nanostructured films on the substrate surface during a SILAR cycle.

Therefore, a basic SILAR cycle comprises four different steps. The steps are represented in the following chart:

Consequently, a SILAR cycle covers four diverse steps on the surface, associating alternative immersion of the substrate into cationic and anionic precursor solution followed by rinsing in each immersion cycle to remove loosely adhered particles as demonstrated in Chart 2 and discussed below:

### 3.1 Adsorption

First SILAR stage forms the Helmholtz double layer owing to the initial adsorption of the cationic precursor such as Cu^+^ on the substrate surface. This layer is generally composed of two charged layers, the positively charged, Cu^+^, inner layer and the negatively charged, (S_2_O_3_)^2−^, outer layers.

### 3.2 Rinsing I

In the second stage, extra adsorbed ions, Cu^+^ and (S_2_O_3_)^2−^, are rinsed away from the diffusion layer towards the bulk solution and a hypothetical monolayer is formed, resulting in a saturated electrical double layer.

### 3.3 Reaction

In the reaction step, the anions, OH^−^, from the anionic precursor solution are entered into the scheme. A solid substance, Cu_2_O, is synthesized on the interface due to the low stability of the material. This procedure pays the reaction of Cu^+^ species with the anionic precursor such as OH^−^.

### 3.4 Rinsing II

In the final SILAR cycle, the extra and unreacted species such as (S_2_O_3_)^2−^, Na^+^ as well as by-products of the reaction from the diffusion layer are removed leaving expected thin films.

**Fig. 3:**
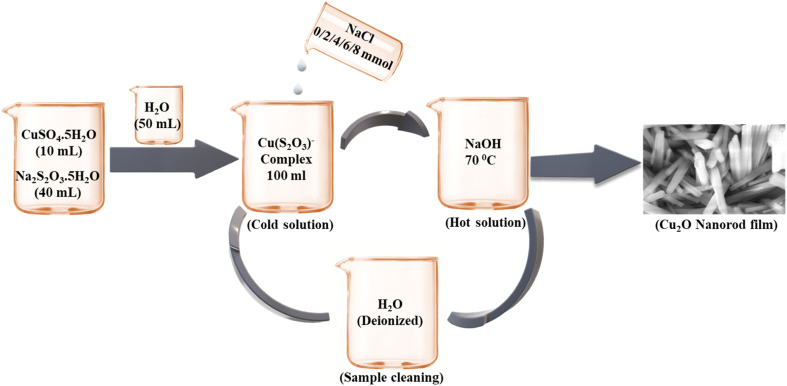
Synthesis of the copper(i) oxide nanorod thin films.^93^

The above deposition process involved alternate immersion of the substrate into cationic and anionic precursor solution followed by rinsing in every immersion cycle to eliminate loosely adhered particles.^36^Fig. 3 represents the synthesis of copper(i) oxide nanorod thin films in presence of NaCl using the SILAR deposition system.^93^ Earlier to the film deposition, the colorless copper–thiosulfate complex was made ready by mixing 10 mL 1 M copper(ii) sulfate and 40 mL 1 M sodium thiosulfate into a 100 mL volumetric flask. Then, in addition to DI water, the required amount of NaCl electrolyte was further added to the same flask and the produced complex solution was the cold solution. Meanwhile, 2 M NaOH solution was kept constant at 70 °C and treated as the hot solution. The substrate such as soda lime glass was then alternatively submerged in cold and hot solutions respectively for the required time interval and completed one SILAR cycle. To fabricate a thin film, this procedure was repeated for up to several immersion cycles.

**Fig. 4:**
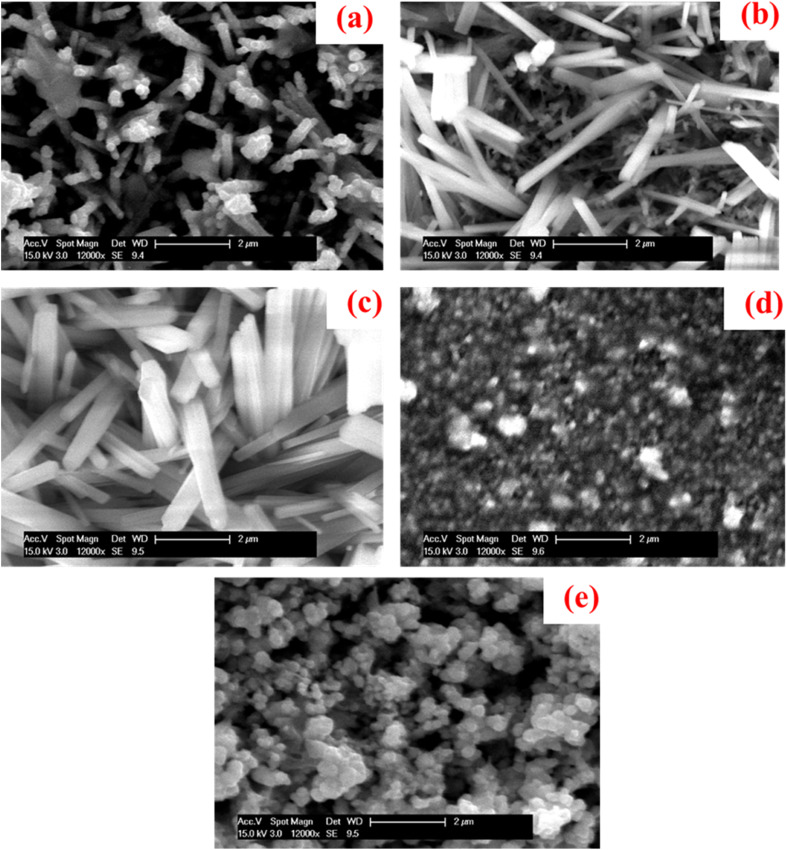
SEM images of the samples fabricated at (a) 0 mmol, (b) 2 mmol, (c) 4 mmol, (d) 6 mmol, and (e) 8 mmol of NaCl electrolyte.^93^

The formation of Cu_2_O nanorod thin films in presence of a NaCl electrolyte at various concentrations were discussed by using SEM micrographs as shown in Fig. 4. The film fabricated with no NaCl electrolyte demonstrated pencil-thin, and crack-free nanorod with an overgrown cluster in some areas on the substrate surface, as also detected in our earlier study.^37^ When 2 mmol of NaCl of the electrolyte was introduced into the solution, the crowded nanorods were developed, and the formation of nanorods enhanced with the increase in the concentration of NaCl to 4 mmol, showing a larger size and shape as observed in the Fig. 4(c). Very rough, tiny and dense spherical grains as well as some overgrown clusters were seen with an additional increase in the concentration of NaCl to 6 mmol. Such an overgrown cluster was produced due to the coalescence of the particles.^94^ Characteristically distributed, clear, and larger-sized spherical grains were revealed with further addition of NaCl electrolyte of 8 mmol. Thus, the NaCl electrolyte has the potential impact to change the surface morphologies from nanorods to spherical grains. The growth of Cu_2_O nanorod thin films was sensitive to the concentration of salts added as also stated for CuO.^95^ The growth of Cu_2_O nanostructures was increased gradually with the rise of NaCl concentration but until a limit. Such phenomena signify that NaCl concentration will consequence in similar morphology of the product and perform key roles in governing the size and shape of the Cu_2_O nanorods. Moreover, the steric hindrance caused by salt concentration might have affected the micelle aggregates, and these effects collected the assemblies of the products. More investigations are ongoing to elucidate the mechanisms for the growth process caused by the novel anticipated route.

## 4. Factors affecting SILAR deposition process

### 4.1 Types of copper salts

In the SILAR technique, Cu_*x*_O thin films were studied and fabricated by using different copper salts as summarized in Table 5. Generally, most of the studies were done by using CuSO_4_·5H_2_O as a basic salt for the formation of Cu_2_O thin films, whereas CuCl_2_·2H_2_O was used for the formation of CuO thin films. The mechanism of both salts was discussed elsewhere in this article.

**Table 5:** Raw materials are used for the formation of Cu_*x*_O thin films by the SILAR method

Formation	Cationic precursors	Complexing agents	Anionic precursors	Ref.
Cu_2_O	Cu(CH_3_COO)_2_·H_2_O	Na_2_S_2_O_3_·5H_2_O	NaOH	96
	CuSO_4_·5H_2_O	Na_2_S_2_O_3_·5H_2_O	NaOH	96
	Cu(NO_3_)_2_·3H_2_O	Na_2_S_2_O_3_·5H_2_O	NaOH	96
	CuCl_2_·2H_2_O	Na_2_S_2_O_3_·5H_2_O	NaOH	96
	CuCl_2_·2H_2_O	NH_3_	H_2_O_2_	97
CuO	CuCl_2_·2H_2_O	NH_3_	H_2_O	98
	Cu(CH_3_COO)_2_·*n*H_2_O	NH_4_CH_3_COO	H_2_O	99

**Fig. 5:**
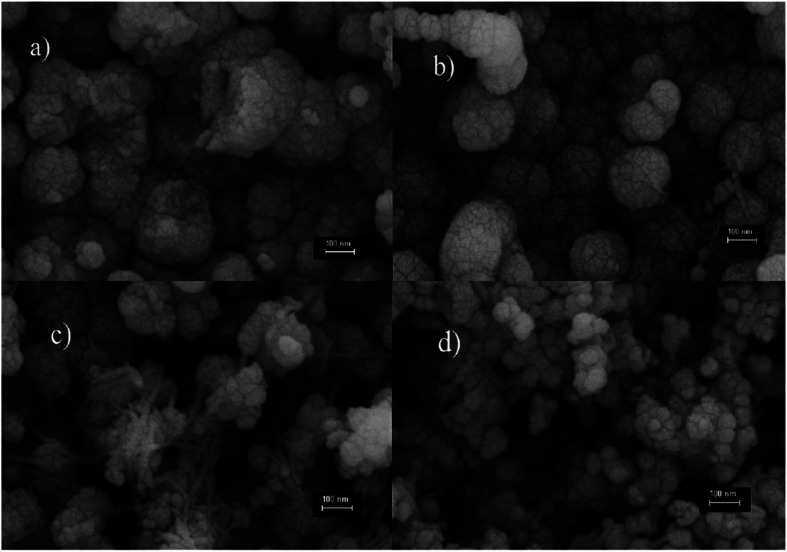
The FE-SEM images of Cu_2_O nanostructured thin films deposited from four different Cu salts^96^ (a) (CH_3_COO)_2_Cu·H_2_O, (b) CuSO_4_·5H_2_O, (c) Cu(NO_3_)_2_·3H_2_O, and (d) CuCl_2_·2H_2_O.

Altindemir *et al.* examined CuSO_4_·5H_2_O with the other three different salts such as (CH_3_COO)_2_·H_2_O, Cu(NO_3_)_2_·3H_2_O and CuCl_2_·2H_2_O to fabricate Cu_2_O thin films. Field emission-scanning electron microscope (FE-SEM) photographs of the deposited Cu_2_O thin films were demonstrated in Fig. 5(a)–(d). The deposited Cu_2_O thin film using the (CH_3_COO)_2_Cu·H_2_O salt demonstrated the cauliflower-like pattern having zero voids between the grains as seen from the images whereas, in the case of the CuSO_4_·5H_2_O salt, the grain size of the film showed the spherical form having no voids between the grains as in Fig. 5(a) and (b). The grain of the Cu_2_O thin film deposited using the Cu(NO_3_)_2_·3H_2_O salt revealed both cauliflower-like and rod shapes as in Fig. 5(c) whereas by means of the CuCl_2_·2H_2_O salt showed the cauliflower-like shape with discrete spaces between the grains as in Fig. 5(d).

On the other hand, CuCl_2_·2H_2_O salt was employed to fabricate CuO thin films using NH_3_ as a complexing agent. But Chatterjee and co-workers utilized H_2_O_2_ as an oxidizing agent with CuCl_2_·2H_2_O and NH_3_ solution to fabricate Cu_2_O thin films instead of CuO thin films.^95^ Similarly, Cu(CH_3_COO)_2_*n*H_2_O and NH_4_CH_3_COO solution could be the potential choice to fabricate CuO thin films.^99^

### 4.2 pH of the precursor solution

Impact of solution pH on the properties of Cu_*x*_O nanostructured thin films deposited by SILAR was studied in the pH scale range from 2.35 to 12 as shown in Table 6. The study was accomplished by controlling the pH of cationic and anionic precursor solutions by adjusting the additional acid and/or bases such as H_2_SO_4_, CH_3_COOH, NaOH and NH_4_OH.

**Table 6:** Properties of Cu_*x*_O thin films deposited by varying solution pH applying SILAR method

pH	Precursors		To alter pH	Phase formed	Thickness (nm)	Crystallite (nm)	Band gap (eV)	Resistivity × 10^3^ (Ω cm)	Ref.
	Cationic	Anionic							
2.35	[Cu(S_2_O_3_)]^−^	2 M NaOH^a^	H_2_SO_4_	Cu_2_O	340	17	2.05	0.21	36
3.45		2 M NaOH^a^	CH_3_COOH		729	21	2.10	0.18	
4.50		2 M NaOH	CH_3_COOH		800	15–22	2.30	72	37
5.10		2 M NaOH	CH_3_COOH		1000		2.28	103	
6.20		2 M NaOH	CH_3_COOH		1800		2.43	742	
7.33		2 M NaOH^a^	—		1130	18	2.15	0.37	36
7.33		1 M NaOH	—		336	13	2.16	0.18	
7.95		2 M NaOH	—		1477	15–22	2.42	21.9	37
9.0	[Cu(NH_3_)_4_]^2+^	H_2_O	—	CuO	42	37.4	1.61	—	105
9.5		H_2_O	—		67	22.4	1.49	—	
10.0		H_2_O	—		85	22.9	1.49	—	
10.0		H_2_O	H_2_SO_4_		520	14	2.17	6.5	94
10.5		H_2_O	H_2_SO_4_		590	21	2.07	5.5	
11.0		H_2_O	H_2_SO_4_		680	27	2.02	4.0	
11.5		H_2_O	H_2_SO_4_		770	30	1.99	4.25	
12.0		H_2_O	H_2_SO_4_		820	36	1.89	4.5	

a OP = optimized precursor (2 M NaOH).

**Fig. 6:**
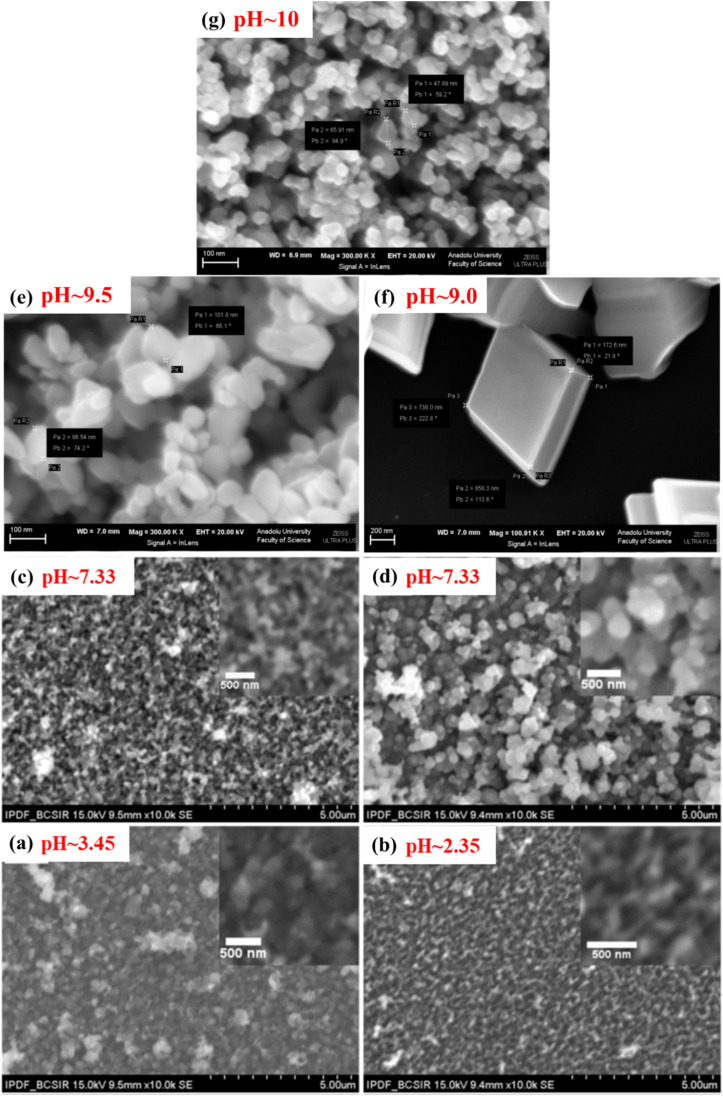
SEM images representing {Cu_2_O: (a)–(d) and CuO: (e)–(g)} thin films deposited by varying pH.^36,105^

To optimize the growth condition to fabricate the FTO/Cu_2_O/ZnO heterojunction solar cell, Farhad and co-workers extensively studied the effect of pH in between 2.35 and 7.95.^36^ During the study, the Cu_2_O thin films have grown by slightly modifying the original SILAR method,^100^ just by eliminating step 2 as shown in Chart 1 and named the technique as modified SILAR method. 10% H_2_SO_4_ and CH_3_COOH were added dropwise into cationic precursor solution to adjust solution pH, as well as the concentration of anionic precursor (NaOH), was also varied (1–2 M). The addition of CH_3_COOH into the optimized precursor solution (OP + CH_3_COOH) improved the quality of the crystal having a larger crystallite size of 21 nm whereas H_2_SO_4_ played the opposite role. Strong H_2_SO_4_ etches film thickness and it decreased with decreasing pH of the cationic precursor solution. From the SEM micrograph it is observed that the optimized solution (OP ∼ 2 M NaOH) showed compacted and larger spherical grains (*D* ∼ 231–259 nm) while the non-optimized solution (1 M NaOH) revealed irregular surface morphology with tiny grains (*D* ∼ 164 nm) which is as shown in Fig. 6. Grain size and band gap decreased with decreasing pH of cationic precursor solution such as 259–164 nm and 2.16–2.05 eV respectively. The electrical resistivity varies in the range of 0.18–0.38 kΩ cm and among optimized solutions OP + CH_3_COOH showed the lowest resistivity. The resistivity of the modified SILAR grown samples had (1–5) order magnitudes less than those deposited by Nair and Ristov's SILAR, and electrodeposition method.^36^

**Fig. 7:**
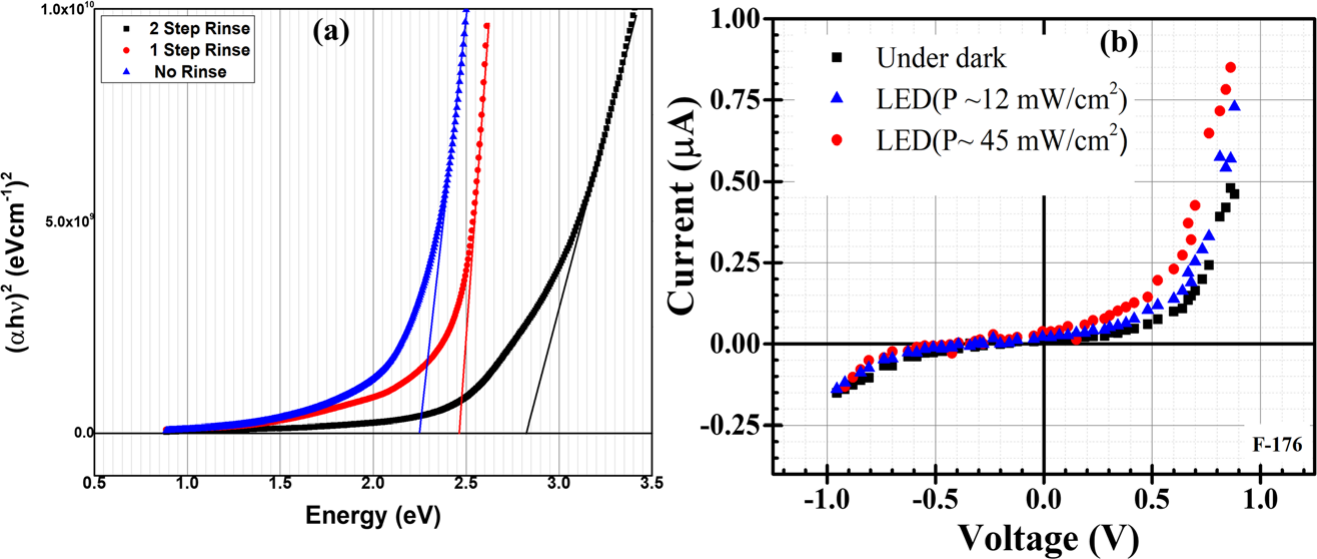
(a) Variation of band gap with rinsing steps. (b) *I*–*V* characteristic curve for FTO/Cu_2_O/ZnO heterojunction cell grown on FTO substrate at pH-7.95.^101^

To justify further the effects of CH_3_COOH and rinsing steps, Farhad and co-workers again deposited thin films at pH 4.50–7.95 by adding CH_3_COOH into cationic precursor solutions.^37,101^ It is seen that film deposited without any rinsing step showed the lowest band gap due to the high film thickness and *vice versa* which is shown in Fig. 7(a). pH 5.10 which was maintained by adding CH_3_COOH exhibited a larger and densely packed grain size (∼300–530 nm) compared to pH 7.95 where no use of CH_3_COOH. Band gap and resistivity values were 2.41–2.30 eV and 742–72 kΩ cm respectively and decreased with decreasing pH of cationic precursor solution.^37^ The activation energy was 0.004–0.19 and 0.01–0.68 eV respectively in the temperature range 40–90 and 100–250 °C. In these studies, FTO/Cu_2_O/ZnO heterojunction shows diode-like characteristics^101^ at pH 7.95 as shown in Fig. 7(b) with maximum power (*P*) of ∼45 mW cm^−2^ under LED illumination with Knee voltage −0.5 V. These results indicate that CH_3_COOH has a significant influence on the controlling of the properties of Cu_2_O thin films.

Umeri and coworkers described the effect of pH and growth temperature during the deposition of Cu_*x*_O thin films^94^ at RT and 70 °C in the pH range from 8 to 11. At RT, the pure Cu_2_O phase exists with pH 8, while mixed phases of Cu_*x*_O appeared at pH 11. Whereas, at 70 °C, only the pure Cu_2_O phase was deposited in the pH region of 8–11, which indicates that 70 °C is the optimum temperature for the growing phase of pure Cu_2_O thin films as also supported by other reported results.^102^ At 70 °C, the optical band gap (*E*_g_) rises from 1.85 to 2.0 eV with the rise of pH from 8 to 11, while the trend showed the opposite at RT and declines from 2.0 to 1.6 eV with the rise of pH. This might be because of the change in the composition from Cu_2_O to CuO. From SEM micrographs, it is seen that at RT, the compact thin film was produced with pH 8 with an overgrown cluster in some spaces and when the pH increased to 11, overgrown cluster formation was diminished, and network-like nanofibers were observed. Conversely, at 70 °C and pH 9, a fiber-like nanostructure was formed, that looked like the morphology of films grown at RT with pH 11. Consequently, uniform, overgrown clusters free of close-packed and interconnected nanofibers of Cu_2_O were observed at 70 °C and pH 11 with a band gap of 2.0 eV. Thus, temperature-dependent pH has a significant controlling overgrowth and properties of the deposited films.

**Fig. 8:**
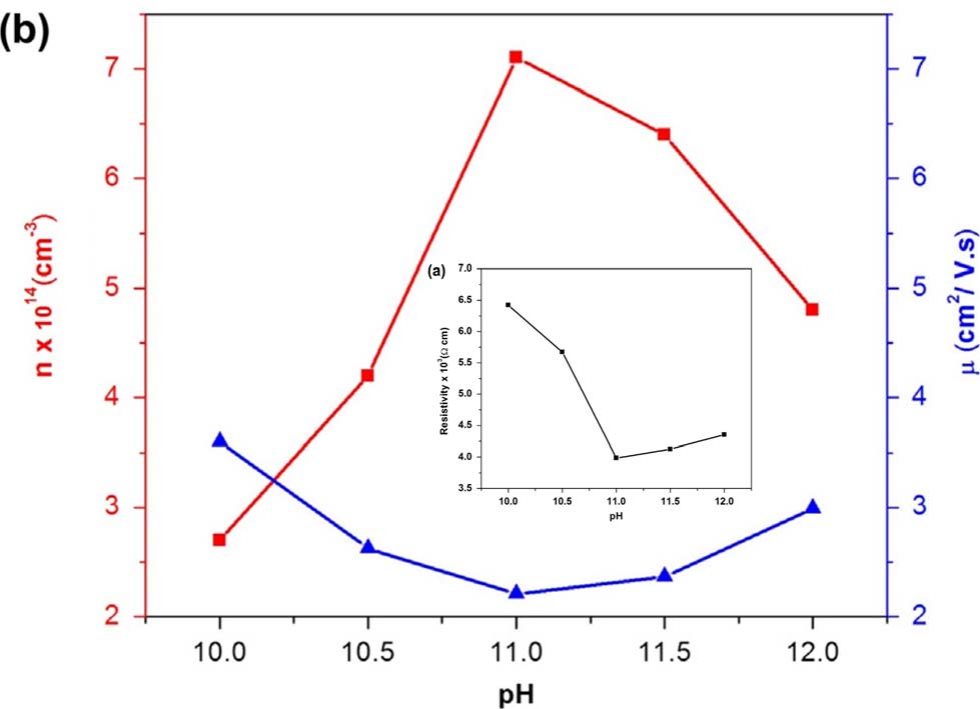
(a) Electrical resistivity, (b) mobility and carrier concentration variation of SILAR-coated CuO thin films with pH.^94,103^

Likewise, Cu_2_O, the influence of pH on the physical properties of CuO thin films was investigated by Visalakshi *et al.*^103^ The pH (∼10–12) of the cationic precursor solution was maintained by adding concentrated NH_4_OH. Film thickness, crystallite size, and texture coefficient rise with the rising pH of cationic precursor solution but dislocation density and strain decreases. SEM images concluded that pH 10 and 10.5 exhibited cluster-like surface morphologies due to the coalescence of the grains but when it reached pH 11, uniformly distributed spherical grains were observed. At pH > 11 the agglomeration of the grains occurs which outcomes in larger grain size. The optical transmittance and band gap (2.17–1.89 eV) reduces with increasing pH. The resistivity decreases initially from 6.5 × 10^3^ to 4.0 ×10^3^ Ω cm with increasing pH from 10 to 11, then further increases with increasing pH. As represented in Fig. 8(a), with the increase of solution pH from 11 to 12, the carrier concentration decreases from 7.1 × 10^14^ to 4.8 × 10^14^ cm^−3^ which is in good agreement with the obtained result by Saravanakannan *et al.*^104^ Conversely, the mobility is first declined to pH 11; then, it is raised for further growth in pH. The decrease in mobility may be owing to the scattering formed at grain boundaries. The decrease in resistivity of the film synthesized at high pH may be attributable to the growth in film thickness without voids, whereas the rise in resistivity and decrease in carrier concentration and mobility detected at low pH may be owing to the existence of bulky voids. However, almost different properties were exhibited when the sample was annealed at 400 °C for 2 hours after deposition reported by Gençyılmaz and co-workers.^105^

### 4.3 Number of cycles during deposition

The film characteristic is closely related to the number of immersions of the substrates into the precursor solution. In almost all studies, the number of cycles is generally kept constant to understand the other properties of the Cu_*x*_O films. There are few investigations where the effect of the number of cycles on the fabrication of Cu_*x*_O films^106–110^ is discussed, as summarized in Table 7. It is obvious that with the increase of immersion cycles thickness of the films increase.

**Table 7:** Effect of the number of cycles on the fabrication of SILAR grown Cu_*x*_O films

Product	Cycles	Thickness (nm)	Crystallite (nm)	Dislocation density (*δ* × 10^−3^, nm^−2^)	Strain (*ε* ×10^−3^)	Bandgap (eV)	Ref.
Cu_2_O	50	550	16	3.72	6.82	2.11	106
	75	830	19	2.90	6.01	2.06	
	100	1050	20	1.51	4.35	1.84	
	10	120	2.46	—	—	2.01	107
	30	350	5.23			2.48	
	40	520	7.15			2.53	
	20	654	—	—	—	2.48	37
	40	1130				2.45	
	60	1200				2.41	
	80	1477				2.38	
CuO	30	500	11	8	3.12	1.56	108
	40	850	13	6	2.66	1.52	
	50	950	18	3	1.95	1.48	
	20	87	7	2.04	3.62	2.48	109
	30	179	8	1.56	3.73	2.41	
	40	298	9	1.23	3.83	2.37	
	50	415	11	0.83	4.87	2.31	
	30	—	10	10	3.43	1.92	110
	40		15	4.44	2.27	1.89	
	50		24	1.74	1.42	1.69	

**Fig. 9:**
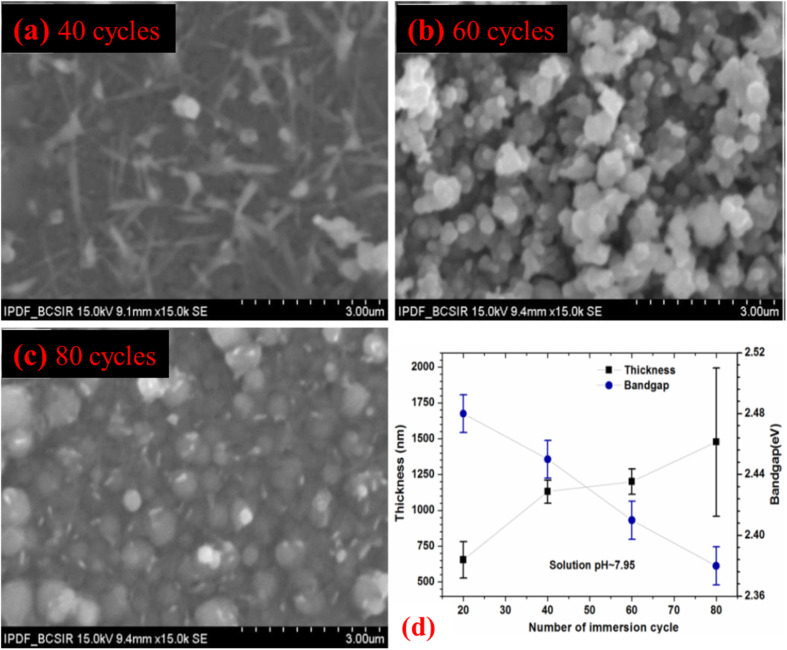
SEM micrographs of the films grown with different immersion cycles at pH: 7.95 for (a) 40 cycles (b) 60 cycles (c) 80 cycles and (d) film thickness of Cu_2_O thin films using different immersion cycles grown by m-SILAR.^37^

The surface SEM morphologies of m-SILAR deposited Cu_2_O films in the top of the FTO substrates using 40, 60, and 80 immersion cycles were shown in Fig. 9(a)–(c). Throughout the area investigated, the surface morphology of all films was seen to be compact as well as coherently carpets. However, the Cu_2_O film grown with 40 immersion cycles demonstrated fiber-like microstructures with small grains of around 200 nm. Instead, thin films having 60 and 80 immersion cycles exhibited bigger spherically shaped grains of size around (200–550) and (350–650) nm respectively as shown in Fig. 9(b) and (c). This reflection recommends that grain size develops as the thickness increases with the increase of immersion cycles.^37^ As can be seen from Table 7 and Fig. 9(d), the optical bandgap of the Cu_2_O thin films deposited at pH ∼ 7.95 using 20 cycles (film thickness ∼ 654 nm), 40 cycles (film thickness ∼ 1130 nm), 60 cycles (film thickness ∼ 1200 nm) as well as 80 cycles (film thickness ∼ 1477 nm) were calculated to be ∼2.48, ∼2.45, ∼2.41 and ∼2.38 eV respectively. Obviously, there is a decreasing trend of optical bandgap with increasing film as represented in Fig. 9(d), probably due to the bigger grains usually existing in the thicker films, which verifies the results stated by Nair *et al.*^111^

**Fig. 10:**
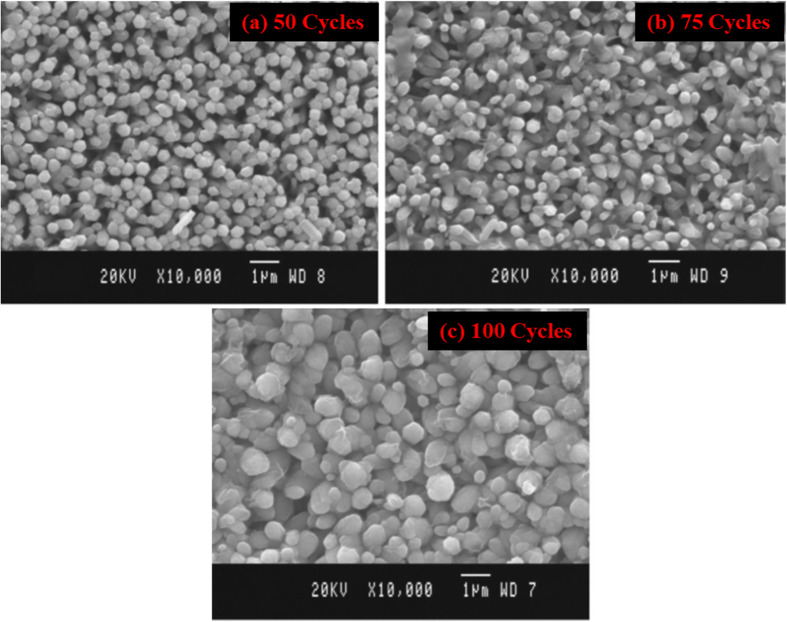
SEM image of the Cu_2_O nanostructured films deposited at (a) 50 (b) 75 and (c) 100 dipping cycles.^106^

**Fig. 11:**
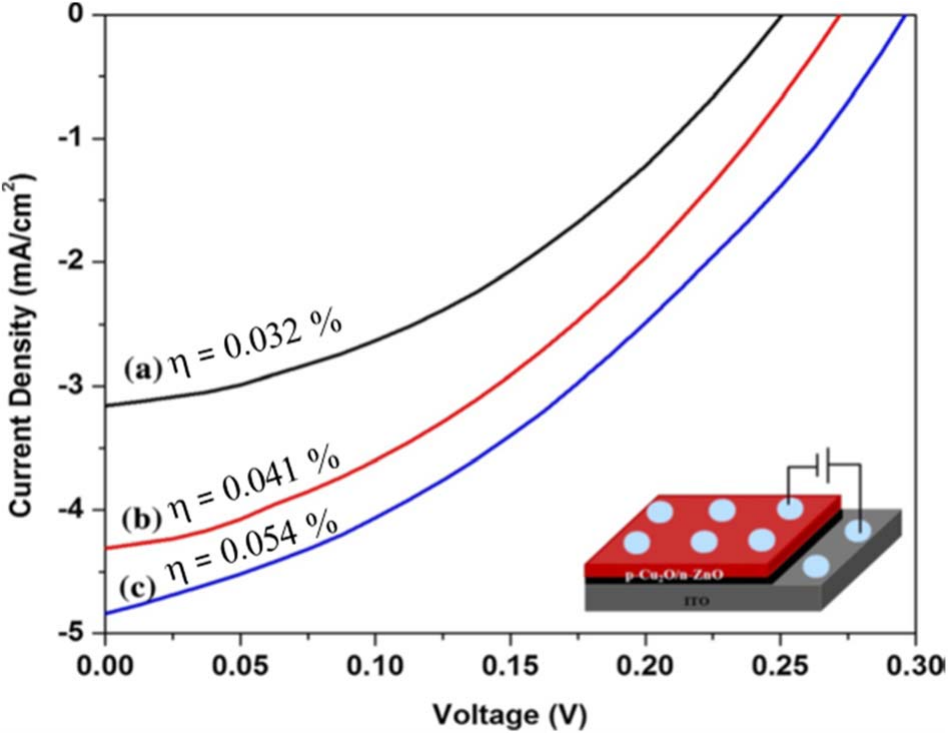
Current–voltage curve of ITO/ZnO NRs/Cu_2_O/Al cells (a) 50 (b) 75 and (c) 100 dip Cu_2_O films.^106^

Fig. 10(a)–(c) demonstrates the Cu_2_O nanostructured films fabricated with different dipping cycles of the nanorods spread homogeneously on the substrate surface, showing a large number of grains with fine particle edges. As seen from morphological studies and Table 7, 50 cycles grown sample has a smaller crystallite size and higher dislocation density with good nanorod morphology. For light absorption, although it has an opportunity for a larger surface area to the photoelectrode, due to the presence of considerable grain boundaries, it creates recombination problems in the film. So, the electron trapping at the surface and in the intergrain boundaries lowered the efficiency value of the film grown through 50 cycles. The samples deposited by 75 and 100 cycles have comparatively better crystallite size and lower dislocation density, which leads to reduce grain boundaries.^106^ Due to the drop in grain boundary resistance, the photogenerated charge carriers can significantly reduce the recombination losses. The cell was fabricated as ITO/ZnO NRs/Cu_2_O/Al with varied efficiency mainly due to the number of cycles of the films shown in Fig. 11. Even though the attained efficiency of the ZnO/Cu_2_O heterojunction was lower, the efficiency was high in the samples deposited at high cycles such as 100. Hence, the effect of the film thickness on cell performance was evidenced by the enhancement of efficiency due to the substantial development of crystallinity and absorbance of Cu_2_O films.

### 4.4 Effect of bath temperature

**Fig. 12:**
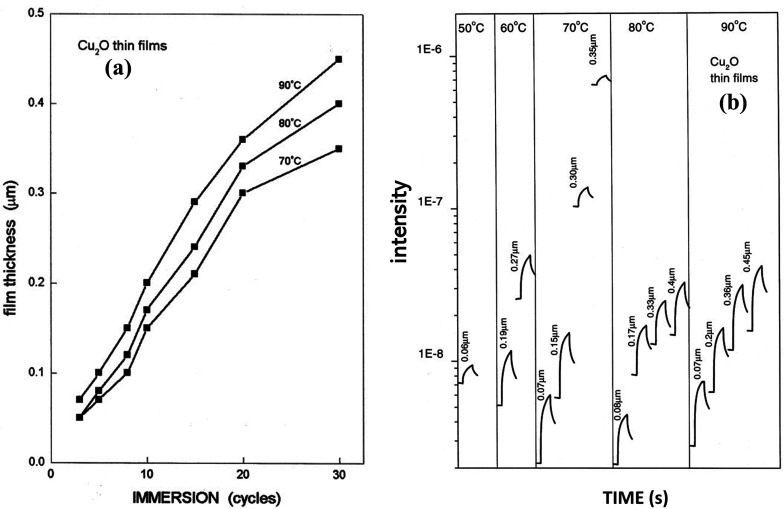
Samples in NaOH solution (a) at different temperatures as a function of film thickness and immersions. (b) At the designated temperatures to record the photocurrent response of the Cu_2_O thin films of different thicknesses.^111^ [Light source: tungsten–halogen, intensity of illumination: 1 kW m^−2^, time length: 60–180 s, bias: 1 V applied across Ag print electrodes 5 mm (long) × 5 mm (separation)].

Fig. 12(a) illustrates the deposition of the thin films grown by varying bath temperatures of anionic precursors as a function of the immersion cycles. The figure demonstrated that the fabrication rate reduced as the immersion cycle proceeds characteristically at 10 nm per cycle. In the case of fabrication using the alkali solution at 90 °C, the production was faster, and the film thickness was >0.3 μm with 20 cycles of immersions, whereas, at 70 °C, the film growth slightly falls after 20 immersions.^111^ For the films fabricated with NaOH solution temperature of 50–90 °C, the photo response curves were given for a range of thicknesses as demonstrated in Fig. 12(b). Irrespective of the solution temperature, the dark current and the photocurrent logged in the films were comparable for the films with thicknesses smaller than 0.1 mm. The values were higher in samples fabricated using NaOH solution at 70 °C having films of higher thickness. The measured electrical conductivity of a 0.15 μm film is about 5 × 10^−4^ Ω^−1^ cm^−1^. And it was found that the increase of film thickness of two orders increases the conductivity by nearly two orders.

**Fig. 13:**
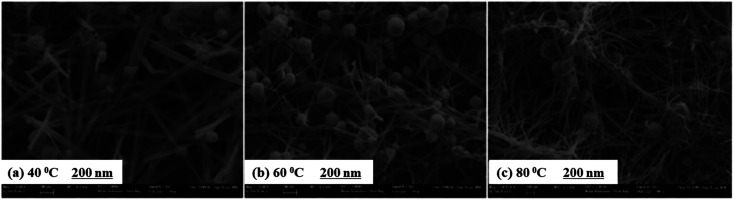
SEM images of Cu_2_O nanostructured thin films deposited by SILAR at different bath temperatures (a) 40 °C, (b) 60 °C, and (c) 80 °C.^112^

The structural parameters, elemental composition, and optical band gap for different bath temperature of Cu_2_O films are given in Table 8, studied by Baig *et al.*^112^ It is seen from the table that when the temperature climbed from 40 to 80 °C, the size of grain increased from 16.78 to 18.84 nm whereas strain in the crystal lattice was reduced. The fall in strain signifies that the imperfection in the crystal lattice with the rising temperature was decreased. The SEM images of Cu_2_O thin films deposited on ITO substrate with different anionic bath temperatures are demonstrated in Fig. 13. From the figures, with the rise of anionic bath temperature the structure of the wire became compact compared to that at 40 °C. Likewise, the oxygen concentration was decreased with an increase in temperature as observed in Energy-dispersive X-ray spectroscopy (EDS) value of Cu_2_O film in Table 8. Further, the photocatalytic activity for water splitting by the deposited Cu_2_O thin films at different temperatures was studied in a photochemical cell and the result revealed that film grown at 80 °C had a higher current ratio with respect to the other two samples and the photocurrent produced by that sample is relatively steady (figure in 5.2 section).

**Table 8:** Structural parameters, elemental composition (EDS), and optical band gap for different bath temperatures of Cu_2_O films

Bath temperature (°C)	*D* (nm)	Dislocation density (*δ*) × 10^14^ m^−2^	Micro strain (*ε*)	Atomic (%)		Band gap (eV)
				Cu	O	
40	16.78	35.51	0.007025	50.75	49.25	2.25
60	17.10	34.19	0.006892	56.45	43.55	2.14
80	18.84	28.17	0.006193	61.76	38.24	2.07

### 4.5 Addition of additives

**Fig. 14:**
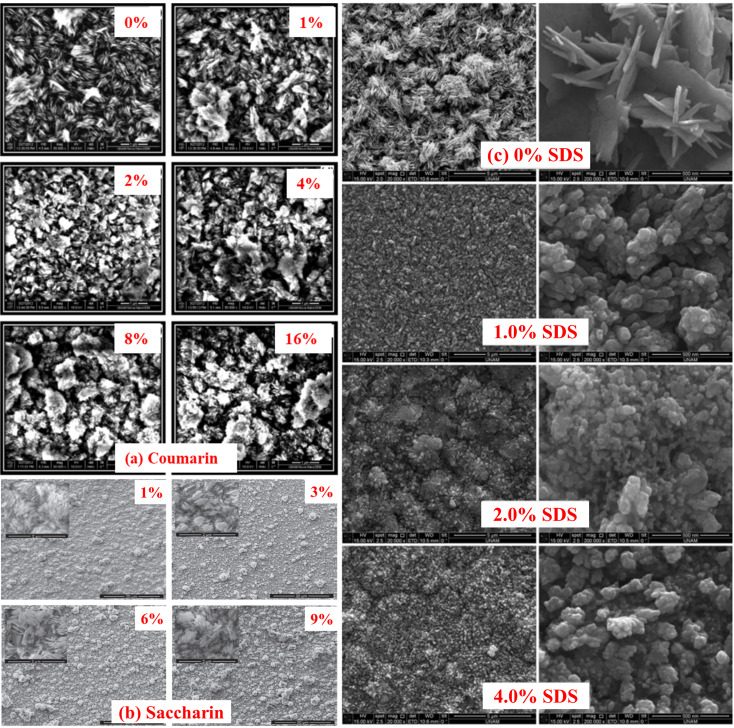
SEM images of CuO nanostructured thin films as a function of different additive concentrations, such as (a) coumarin,^115^ (b) saccharin^116^ and (c) SDS.^117^

The influence of the different additives on the surface morphological characteristics of CuO films was studied by using SEM. Fig. 14 illustrates the SEM images of the CuO thin films fabricated in the solution containing additives such as coumarin, saccharin, and sodium dodecyl sulfate (SDS) having different concentrations. In the first step without coumarin, Fig. 14(a) there were plate-like CuO nanostructures that homogenously cover the entire surface. Then, the nanostructures start to change their shapes with the increase of coumarin concentration, form some clusters on the surface and lose their homogeneity. From Fig. 14(b), in the case of saccharin, it was observed that all the samples have nearly the same morphology that is they are all composed of plate-like nanostructures as in ref. 113. On the other hand, the homogeneity of the films deteriorates with increasing saccharin concentration. In the case of SDS, Fig. 14(c) demonstrates that the fabricated CuO thin films were adhered and spread well onto the substrate surface without SDS. With the addition of SDS and its concentration, the surface morphology of the CuO film was changed dramatically. From this viewpoint, the discrepancy of SDS molar concentration impressed the morphology of the surface of all the fabricated thin films. This variation in morphology may be owing to the electrostatic interaction between Cu^2+^ and CH_3_(CH_2_)11OSO_3_^−^. SDS may affect particle growth as well as morphology after nucleation. Thus, during the crystallization process, the SDS can affect nucleation.^114^

Through UV-Vis's spectrophotometer study, it was clear that both the optical band gap and the transmission spectra were affected by the additive concentration. The band gap, as well as spectral transmittance values of the films, were decreased for the higher content of both coumarin and saccharin,^115,116^ while showing the opposite tendencies in the case of SDS. The optical bandgap energy of both organic (coumarin and saccharin) additives decreased from around 1.50 to 1.27 eV, while increased from 1.32 to 1.49 eV for inorganic SDS, with the increasing concentration of the additives.^117^

### 4.6 Complexing agent

**Fig. 15:**
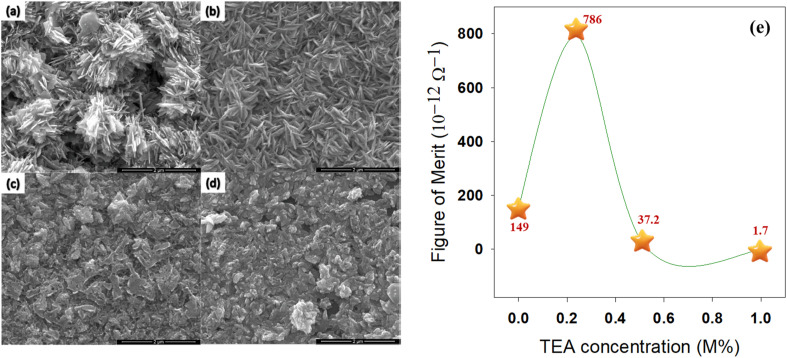
SEM images of CuO thin films at (a) 0 M%, (b) 0.25 M%, (c) 0.50 M%, (d) 1.00 M% as well as (e) FOM with TEA at different molar concentrations.^118^

Cavusoglu and co-workers studied the role of the complexing agent such as triethanolamine (TEA) mediated fabrication of nanocrystalline CuO thin films *via* SILAR technique at room temperature and the results are summarized in Table 9. As a function of increasing TEA concentration, the optical band gap energy of the fabricated CuO thin films was increased from 1.33 to 2.00 eV while the average transmittance of all the films increased from 2.5 to 42.5%. A minimum resistivity of 3.74 × 10^5^ Ω cm was found with zero TEA in CuO thin films whereas, with a TEA concentration of 1.0 M%, the resistivity subsequently increased to 509 × 10^5^ Ω cm. Surface morphology on the film surfaces demonstrated the homogeneous distribution of the nanostructured CuO as demonstrated in Fig. 15(a)–(d) whereas, the figure of merit (FOM) was represented as a function of TEA concentration as shown in Fig. 15(e). TEA concentration of 0.25 M% in CuO thin film provided the high FOM values of 786 × 10^−12^ Ω^−1^ at distinct wavelengths of between 600 and 900 nm.^118^ Therefore, the range of optical and electrical properties developed by such a study having a different complexing agent is favorable for the applications of numerous optoelectronic devices.

**Table 9:** Properties of CuO thin films as a function of TEA concentrations

TEA concentration *M*%	Crystallite (nm)	Thickness (nm)	Bandgap (eV)	Conductivity (*σ*) ×10^−6^ (Ω cm)^−1^	Resistivity (*ρ*) ×10^5^ Ω cm	FOM × 10^−12^ Ω^−1^
0	19.95	797	1.33	2.67	3.74	149
0.25	19.80	387	1.57	1.05	9.50	786
0.50	18.92	199	1.67	0.07	149	37.2
1.00	17.47	101	2.00	0.02	509	1.70

### 4.7 Annealing of as-deposited films

Annealing is a vital parameter to control the phases of the deposited thin films. Both the phases of Cu_*x*_O could be synthesized by changing the atmospheric condition (air, vacuum) and temperature of annealing as summarized in Table 10. Here, air or oxygen^37,101,119–124^ annealing of the SILAR-grown films has been studied more extensively than vacuum annealing.^125^ Recently, SILAR deposited Cu_*x*_O films are mainly studied between 75 to 500 °C^37,101,119–124^ in presence of air or N_2_. The study revealed that Cu_2_O phase was stable until 250 °C,^120,122^ though Farhad and co-workers showed a mixed phase of both Cu_2_O and CuO at 250 °C^37^ due to the pH effect, while Ozaslan *et al.* showed a mixed phase even at 500 °C.^123^ The CuO phase could be found at 300 °C^120^ by annealing of Cu_2_O or even could be deposited by using NH_3_ solution (pH = 10) with the reaction of CuCl_2_ at ambient temperature.^124^

**Table 10:** Annealing of the SILAR grown Cu_*x*_O nanostructured thin films

Anionic salt, NaOH (M)	Cycles	Time		Temperature		Crystal phase	Crystallite (nm)	Band gap (eV)	Ref.
		Dipping (s)	Annealing (min)	Growth (°C)	Annealing (°C)				
**CuSO**_**4**_ **·5H**_**2**_ **O + NaOH + Na**_**2**_ **S**_**2**_ **O**_**3**_ **·5H**_**2**_ **O**									
0.5	20	20	—	70	200–400	As deposited: Cu_2_O	27.76	—	119
						200 °C: Cu_2_O	49.95		
						300 °C: Cu_2_O + CuO	40.88		
						400 °C: CuO	62.32		
1	10	20	60	70	200–350	As deposited: Cu_2_O	14	2.20	120
						200 °C: Cu_2_O	14	2.20	
						250 °C: Cu_2_O	14	2.20	
						300 °C: CuO	14–26	1.35	
						350 °C: CuO	—	1.35	
1	30	20	—	70	200–400	As deposited: Cu_2_O	14–26	2.40	121
						200 °C: Cu_2_O		2.40	
						300 °C: Cu_2_O + CuO		2.06	
						400 °C: CuO		1.73	
1	30	20	—	50–90	250–400 (air, N_2_)	As deposited: Cu_2_O	∼18	2.10	122
						250 °C: Cu_2_O		2.10	
						300 °C: Cu_2_O + CuO		—	
						350 °C: CuO		1.75	
						400 °C: CuO		1.75	
2	40	2	60	70	100–500	As deposited: Cu_2_O	—	2.57	123
						100 °C: Cu_2_O		2.52	
						300 °C: Cu_2_O (2.27%) + CuO (97.73%)		2.45	
						500 °C: Cu_2_O (1%) + CuO (99%)		1.91	
2	40–80	—	60–180	70	75–350	As deposited: Cu_2_O	15–22	2.42	37
						75 °C: Cu_2_O		2.02	
						150 °C: Cu_2_O		1.98	
						200 °C: Cu_2_O		1.94	
						250 °C: Cu_2_O + CuO		1.62	
						350 °C: Cu_2_O + CuO (1 h)		—	
						350 °C: CuO (3 h)		1.44	
2	60	5	60	70	350	As deposited: Cu_2_O		2.06–2.16	101
						350 °C: Cu_2_O + CuO (1 h)		1.43–1.51	

CuCl_2_ + NH_3_OH + H_2_O_2_									
—	2–10	30	30	RT	20–500	As deposited: Cu_2_O	14	2.17	124
						100 °C: Cu_2_O	14	2.22	
						150 °C: Cu_2_O	14	2.17	
						450 °C: CuO	16	1.43	
						500 °C: CuO	16	1.44	
—	400	>30	60	RT	27–600 (air, vacuum)	As deposited: Cu_2_O	14–21	2.30	125
						100 °C: Cu_2_O		2.40	
						400 °C: CuO		1.85	
						600 °C: CuO		1.70	

**CuCl**_**2**_ **+ NH**_**3**_ **solution (pH = 10)**									
—	80	30	30	—	200–400	As deposited: CuO	11.09	1.17	126
						200 °C: CuO	12.05	1.29	
						300 °C: CuO	13.86	1.30	
						400 °C: CuO	14.88	1.36	

**Fig. 16:**
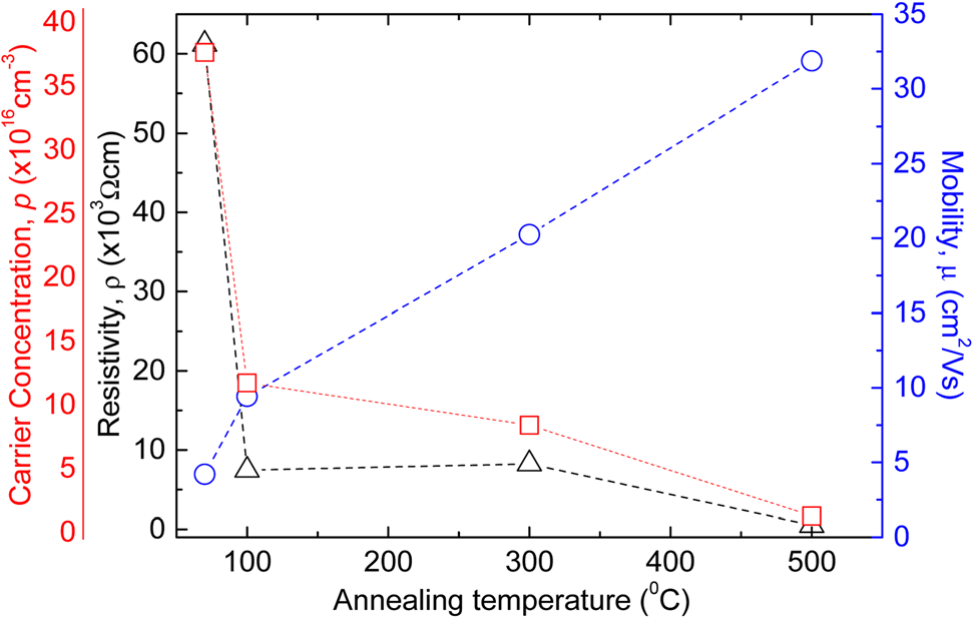
Representation of the change of resistivity, mobility, and carrier concentration of copper oxide (Cu_2_O and CuO) films with annealing temperature.^123^

Amudhavalli and co-workers successfully showed the increasing trend of the crystallite size of copper oxides with the increase of annealing temperature while depositing the films at 0.5 M NaOH. Fig. 16 demonstrated the change of resistivity, mobility, and carrier concentration of copper oxide (Cu_2_O and CuO) phases with annealing temperature as shown by Ozaslan *et al.*^123^ It is found that the carrier concentration was decreased from 3.07 × 10^17^ to 6.61 × 10^15^ cm^−3^ with increasing annealing temperature from 70 to 500 °C respectively. The hole mobility of the films was increased from 4.20 to 31.87 cm^2^ V^−1^ s^−1^ with decreasing the carrier concentration, while the electrical resistivity of the films decreased with annealing temperature, inducing the increment in the conductivity of the films. Nair *et al.* observed the dark conductivity of the CuO film produced by air annealing of a 0.15 μm Cu_2_O film at 400 °C is high about 7.2 × 10^−3^ Ω^−1^ cm^−1^.

### 4.8 Doping by diverse dopants

Tuning of the structural, electrical, and optical properties of SILAR-deposited Cu_*x*_O films through Fe, Eu, Zn, Co, B, Mg, Ni, and Pb doping has been reported extensively by several authors. The summary of the effect of doping on SILAR-deposited Cu_*x*_O films is represented in Table 11. Interestingly, in the case of Cu_2_O film fabrication, the reactants were the same except for the doping materials such as Fe, Eu, Zn, and Co.^127–130^ Similarly, during Co, B, Mg, and Pb doping into CuO films,^131–134^ the reactants were also the same except in the case of Ni doping.^135^

**Table 11:** Cu_*x*_O nanostructured materials grown in presence of different dopants with their properties

Product	Dopant		Cycle	Crystallite (nm)	Grain (nm)	Bandgap (eV)	Ref.
	Material	Amount					
**Reactants: CuSO**_**4**_ **·5H**_**2**_ **O + NaOH + Na**_**2**_ **S**_**2**_ **O**_**3**_ **·5H**_**2**_ **O**							
Fe: Cu_2_O	FeSO_4_ (wt%)	0	30	62.83	—	1.80	128
		1		59.80		2.10	
		2		41.83		2.36	
		5		36.40		2.45	
Eu: Cu_2_O	Eu(NO_3_)_3_·5H_2_O (at%)	1	100	27	—	2.08	129
		3		24		2.26	
		5		21		2.41	
Zn: Cu_2_O	ZnSO_4_ (wt%)	0	50	18	—	2.34	130
		1		30		2.35	
		2		39		2.37	
		3		52		2.38	
		5		69		2.41	
		10		44		2.39	
Co: Cu_2_O	CoSO_4_ (wt%)	0	30	62.83	—	1.94	127
		1		53.30		2.03	
		2		48.47		2.12	
		5		39.24		2.18	
		10		28.44		2.47	

**Reactants: CuCl**_**2**_ **·2H**_**2**_ **O + H**_**2**_ **O + NH**_**3**_							
Co: CuO	CoCl_2_·6H_2_O (at%)	0	10	22.7	70	1.53	132
		0.5		15.7	44	1.47	
		1		13.6	42	1.45	
		2		13.1	36	1.41	
		3		12.6	32	1.38	
		4		12.2	38	1.36	
B: CuO	H_3_BO_4_ (at%)	0	10	12.9	45	1.52	131
		1		13.1	42	1.48	
		2		14.2	38	1.43	
		3		15.9	30	1.39	
Mn: CuO	Mn(NO_3_)_2_ (at%)	0	10	—	9.94	1.42	133
		1			7.83	1.98	
		3			8.21	2.08	
		5			9.76	2.20	
Pb: CuO	Pb(NO_3_)_2_ (at%)	0	10	—	9.94	1.43	135
		1			17.22	1.80	
		2			16.21	1.76	
		4			15.79	1.72	
		8			13.07	1.68	
		16			8.98	1.65	

**Reactants: Cu(CH**_**3**_ **COO)**_**2**_ **·*n*H**_**2**_ **O + H**_**2**_ **O + NH**_**4**_ **CH**_**3**_ **COO**							
Ni: CuO	Ni(CH_3_COO)_2_·*n*H_2_O		30	—	10–15	—	134

In the case of Co or Fe doping, the crystallite size of the films of Cu_2_O decreased between 62.83 and 28.44 nm when the concentration of the doped material increased gradually, whereas it showed an opposite trend in the case of Zn doping. Interestingly, Fe, Eu, Zn and Co doping into Cu_2_O rises the bandgap of the material a little in every case. On the other hand, in the case of Co and B doping into CuO films, the bandgap deceased while it was increased for Mg and Pb doping.

**Fig. 17:**
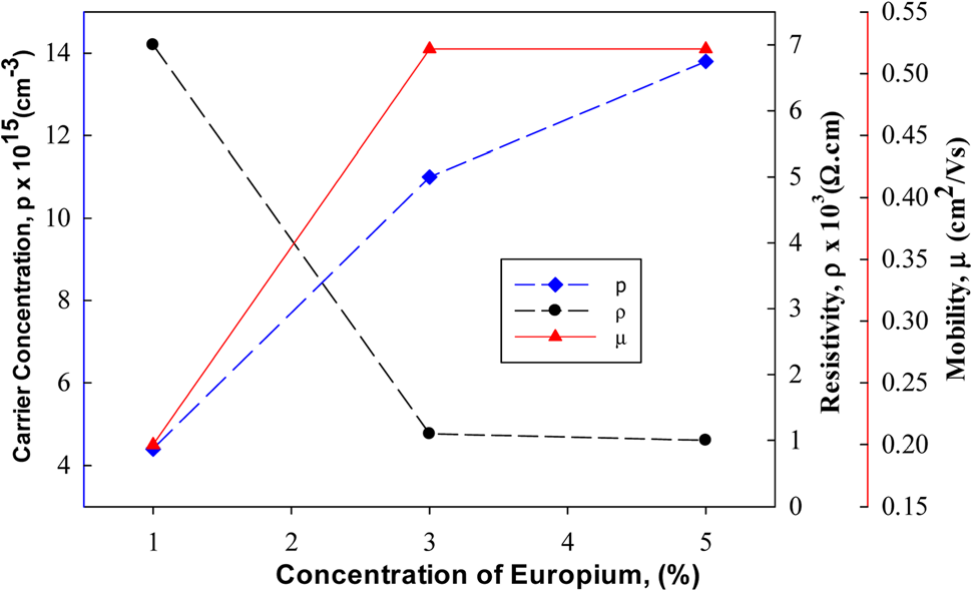
Resistivity, carrier concentration and mobility for Eu doped Cu_2_O films.^129^

The films prepared at high doped Cu_2_O thin films such as 5% Eu showed a low resistivity value of 1 × 10^3^ Ω cm as shown in Fig. 17. The Hall mobility and carrier concentration values in such cases are 0.52 cm^2^ V^−1^ s^−1^ and 13.8 × 10^15^ cm^−3^, respectively.

**Fig. 18:**
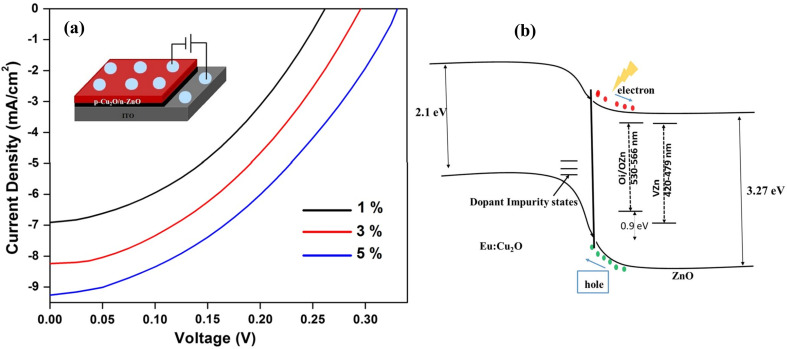
(a) 1%, 3% and 5% Eu doped thin films having current–voltage characteristics of ITO/ZnO NRs/Eu: Cu_2_O/Al cell, (b) carrier transport and band structure of p–n junction.^129^

Fig. 18(a) shows the current density–voltage (*J*–*V*) characteristics of the ZnO/Cu_2_O heterojunction solar cells prepared using the Eu-doped Cu_2_O thin films. The *V*_oc_ was increased with increasing Eu content from 265 mV (1% Eu) up to 332 mV (5% Eu). The conversion efficiency can be enhanced by dropping recombination centers avoiding lattice-mismatch defects, and by reducing the resistance of Cu_2_O. The ionic radius of Eu^3+^ ion was 0.109 nm whereas, Cu^+^ is 0.077 nm. Therefore, Eu^3+^ ion could not be incorporated by substitution rather it was incorporated as an interstitial creating getter center. It overwhelms the recombination losses and thus advances current levels and improved Eu doping levels.^136^

Fig. 18(b) illustrates the band structure as well as carrier transport of the deposited p–n junction. As there was much difference between conduction and valence band off-sets triggering effective separation of charge carriers, a built-in potential barrier was developed. When the light was absorbed onto the device photocarriers were generated and drifted to the respective electrodes depending upon the applied potential causing current conduction. As an acceptor dopant, impurity levels of Eu were adjacent to the valence band edge. In the case of ZnO, the green luminescence at 535 nm could be produced by the diffused Cu ion and replacing Zn. The V_O_ center was atop the valence band whereas the Zn vacancy was (V_Zn_) in an acceptor level, which occurred at 0.8 eV. Nevertheless, the ZnO coated over Eu: Cu_2_O performed as a passivation layer improving the V_oc_ and declining the consequence of impurity center-mediated recombination loss.^137,138^

**Fig. 19:**
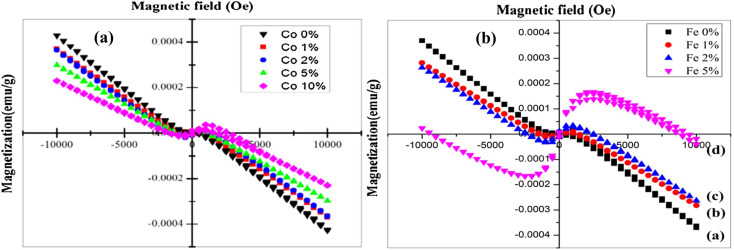
Magnetic behavior of (a) Co-doped^127^ (b) Fe-doped Cu_2_O thin films.^128^

Magnetic measurements were performed by employing a vibration sample magnetometer (VSM) at ambient temperature for both Fe and Co-doped Cu_2_O. Undoped Cu_2_O has a diamagnetic property.^139^ The outcome agrees with Fig. 19(a) and (b) which demonstrate the change of magnetization against the applied magnetic field (*M*–*H*). In the case of Co-doped Cu_2_O, undoped and minimum doped such as 1 and 2 wt% films showed diamagnetic (high magnetization) behavior whereas, at the maximum doped such as 10 wt%, the films showed ferromagnetic (low magnetization) properties.^140^ The diamagnetic order was Cu_2−*x*_Co_*x*_O (*x* = 0 > 1 > 2 > 5 > 10 wt%). The ferromagnetic behavior was possibly due to the intrinsic coupling (Co–Co) between the atoms of doped material. Similarly, in the case of 1% Fe doped Cu_2_O at 305 K, the film showed diamagnetic properties. An increase of the Fe-doping (2 wt%), slightly altered the diamagnetic property because of the increased hole concentrations and further doping of Fe ions (5 wt%), the film showed anti-ferromagnetic behavior. With the increase in the concentration of Fe, both the number of Fe^3+^–Cu^2+^ pairs and the hole concentrations increased and consequently, the crystallite size reduced.

**Fig. 20:**
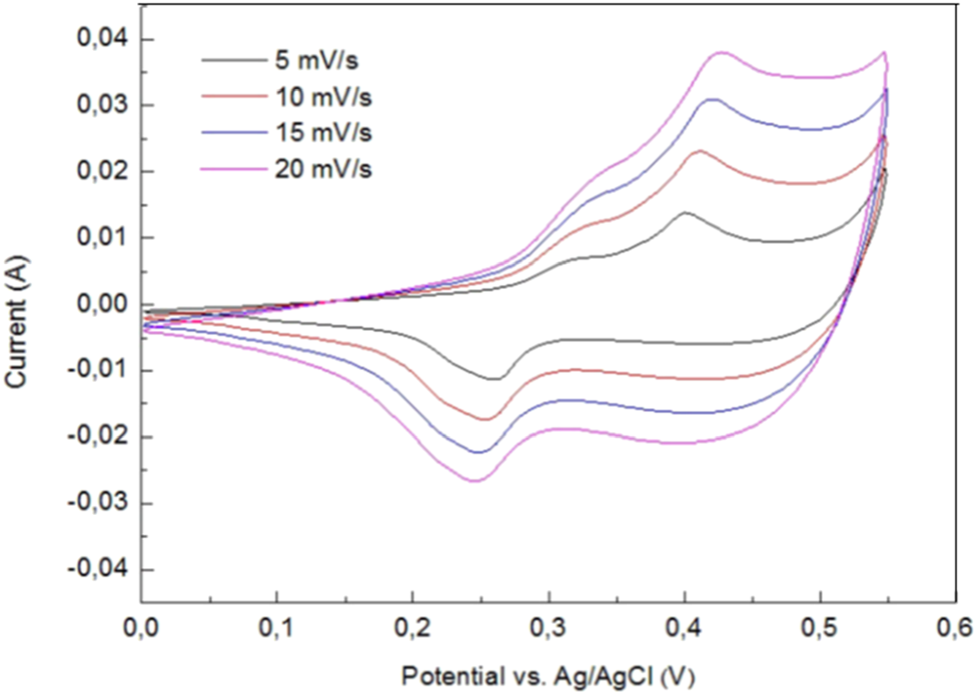
CVA curves for nickel foam electrode with Ni-doped CuO nanolayers at a scan rate of 5, 10, 15 and 20 mV s^−1^.^135^

Lobinsky and co-workers studied the cyclic voltammograms of the nickel foam electrode with Ni-doped CuO nanolayers in a potential space between 0 and 550 mV *vs.* Ag/AgCl electrode at the scanning rates of 5, 10, 15 and 20 mV s^−1^ as shown in Fig. 20. Two of the redox reactions on the anodic curve took place in the layer, including the Cu^+^ → Cu^2+^ transformation at 310 mV while the Ni^2+^ → Ni^3+^ at 390 mV at a scan rate of 5 mV s^−1^. The proportionality of currents to scan rate delivers data that the film is sufficiently thick, and the charge transfer rate was restricted by the diffusion of charge carriers in the film.^134^

**Fig. 21:**
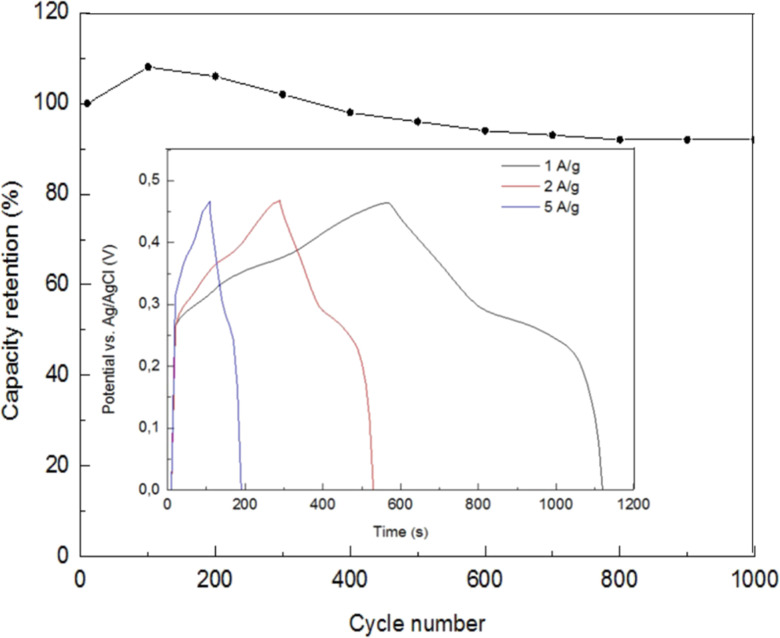
The cycling stability for the Ni Foam electrode with Ni-doped CuO nanolayers at 2 A g^−1^ whereas the inset graph represents galvanostatic charge–discharge curves of the electrode with Ni-doped CuO nanolayers.^135^

Inset of Fig. 21 demonstrates the specific capacitance of the Ni-doped CuO nickel foam electrode, which was found from charge–discharge curves, and it was 154 mA h g^−1^ (1240 F g^−1^) at the current densities of 1 A g^−1^.^135^ The high value of the specific capacitance of the sample can be explained based on the good conductivity of CuO and the substantial role of Ni atoms in pseudo-capacity. The capacity retention of the Ni foam electrode with Ni-doped CuO nanolayers after 1000 charge–discharge cycles at a current density of 2 A g^−1^ was retained at 92%, showing good cycling stability of the material as presented in Fig. 21. High cycling stability could be described by the feature morphology of ultrathin nanocrystals of CuO which deliver fast diffusion of ions on the electrode surface and while not being ruined in the charge–discharge process.

## 5. Applications

The optoelectronic properties of SILAR synthesized thin films have shown outstanding performance in diverse applications, for instance, photovoltaics,^141^ supercapacitors,^142,143^ photoelectrochemical water splitting,^144^ gas sensors^143,145^ and many more. The method appears to be easier and represents an efficient way to manufacture devices. Some of the potential applications such as antibacterial activities, supercapacitors, surface wettability and photoelectrochemical water splitting in presence of Cu_*x*_O nanostructured thin films will be discussed in the following section.

### 5.1. Antibacterial activities

**Fig. 22:**
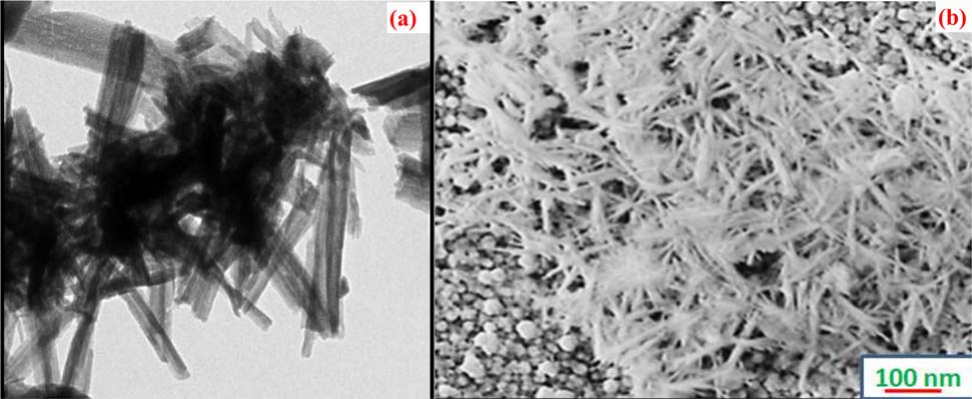
(a) TEM (b) SEM images of the Cu_2_O grains showing needle-shaped uniformity on the surface.^146^

To control pathogens, nanoparticles are in great demand due to their huge applications in the health industries. Results achieved from nanocrystalline Cu_2_O nano-thin films fabricated by Dhanabalan *et al.* possessed substantial antimicrobial activity against the experienced human pathogen at a maximum inhibition zone of 16 mm against Gram-positive *Staphylococcus aureus*.^146^ The surface morphological studies exhibited that the needle-shaped grains which play a crucial role in the antibacterial activity of the fabricated Cu_2_O films by SILAR technique as shown in Fig. 22(a) and (b). The synthesized Cu2O thin film can exhibit antibacterial activity from 18 to 24 hours of incubation time. The bacterial growth will decrease with the increase in the concentrations of nanoparticles, which may be the cause of the reduction of voids affording space for the growth of bacteria that remains resistant to the pathogenic bacterial strain.

### 5.2. Water splitting

**Fig. 23:**
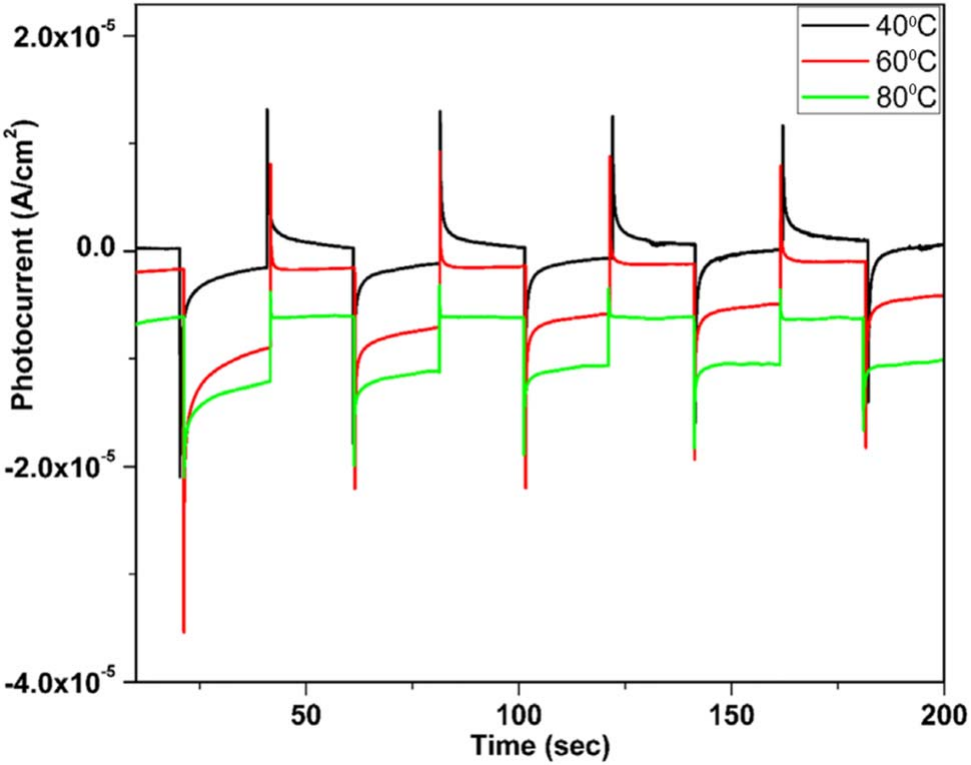
PEC measurement of Cu_2_O thin films at different bath temperatures.^111^

Cu_*x*_O was considered a good candidate for photoelectrochemical (PEC) water splitting due to its abundance, low price, and high stability in aqueous solution.^147,148^ Baig *et al.* fabricated Cu_2_O at a high bath temperature of 80 °C by SILAR which showed high photocurrent and good stability^111^ as discussed earlier. The photocatalytic activity for water splitting of the Cu_2_O thin film was studied with the photochemical system containing Pt (counter), Ag/AgCl (reference), Cu_2_O (working electrode) and KCl (pH = 13.6) as electrolytes. PCE data shown in Fig. 23 revealed that samples synthesized at 80 °C have a higher current ratio and produced a stable photocurrent compared to the other samples by using a 300 W Xenon lamp (PLSSXE300/300UV).

### 5.3. Surface wettability study

**Fig. 24:**
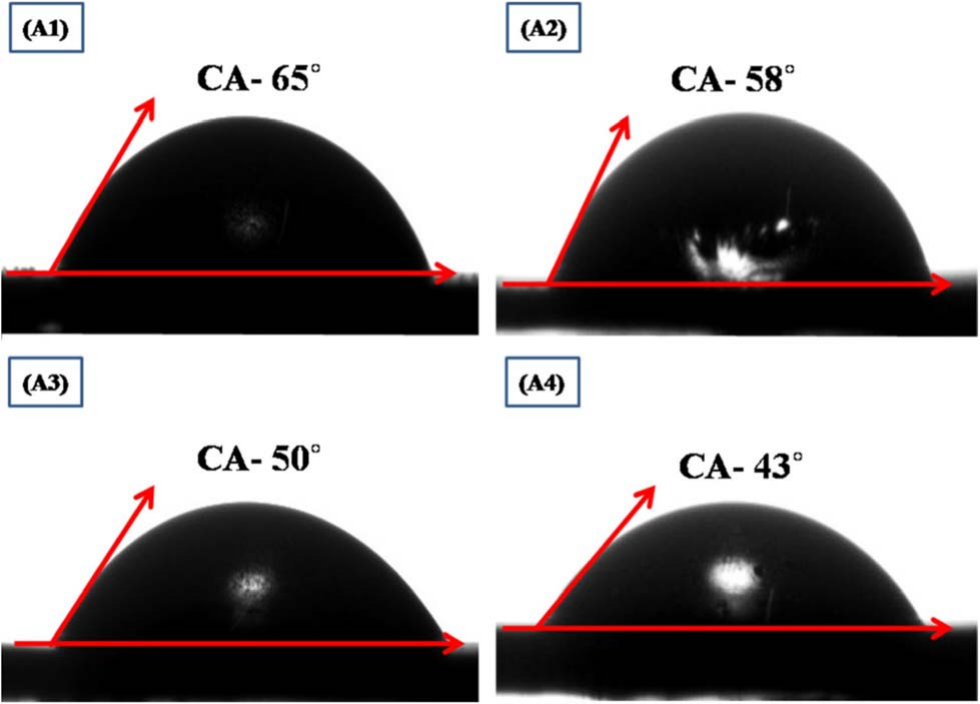
Study of surface wettability of CuO thin films,^149^ (A1) 50 SILAR cycles, (A2) 60 SILAR cycles, (A3) 70 SILAR cycles and (A4) 80 SILAR cycles.

The surface wettability study of films determines its capability to interact with ions when immersed into electrolyte by measuring the contact angle with liquid electrolyte as shown in Fig. 24.^149^ If the contact angle is >90°, then the film surface is hydrophobic, while for <90°, it is hydrophilic. For better interaction of electrolyte ions, the contact angle must be as low as possible with the electroactive site on the thin film surface.^150,151^Fig. 24 (A1, A2, A3, and A4) signifies the image of the contact angle with the surface of the film. The observed angles of CuO thin films with 50, 60, 70 and 80 SILAR cycles were 65°, 58°, 50°, and 43°, respectively. The observed CuO films were hydrophilic in nature, as the contact angles for CuO decline with the rise in SILAR cycles, which will allow more interaction of electroactive sites of the CuO on the film surface.

### 5.4. Super capacitive behavior

**Fig. 25:**
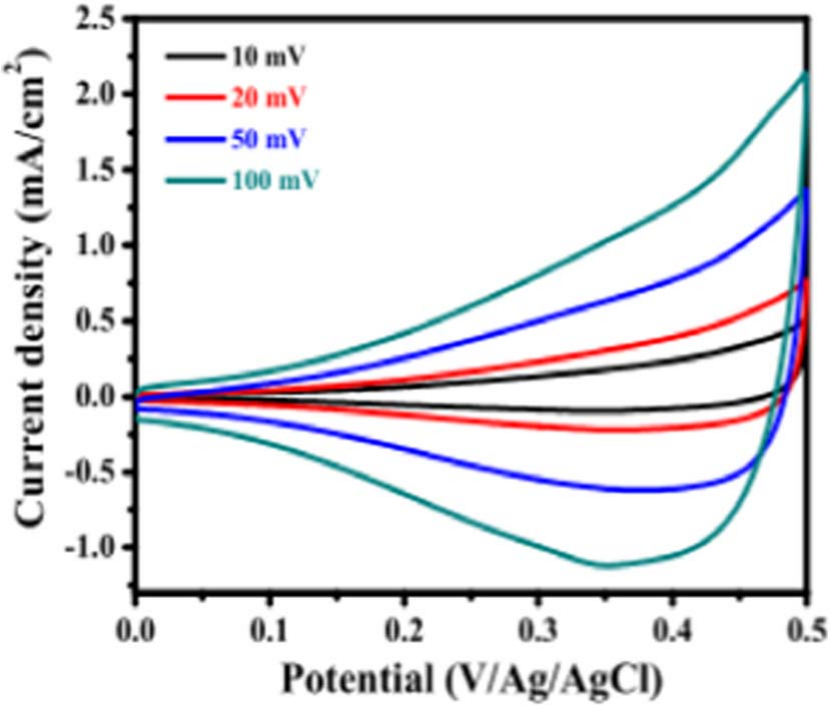
Cyclic voltagramms of CuO with various scan rates of 1 M KOH electrolyte.^149^

The electrochemical impedance, as well as super capacitive properties, of SILAR synthesized CuO thin films are studied by Patil *et al.*^149^ The synthesized CuO thin film showed the lowest charge transfer resistance of 41.45 Ω cm^−2^ with the highest specific capacitance of 184 F g^−1^ at the scan rate of 50 mV s^−1^ and demonstrated 83% capacitive retention after 5000 cycles. Super capacitive performance of the film was verified using cyclic voltammetry (CV) in 1 M KOH electrolytes in a three-electrode cell equipped with CuO (working electrode), Pt (counter electrode) and saturated Ag/AgCl (reference electrode). As shown in Fig. 25, the CVs were studied with a potential window of 0 to 0.6 V/Ag/AgCl at several scan rates such as 10, 20, 50 and 100 mV s^−1^.

**Fig. 26:**
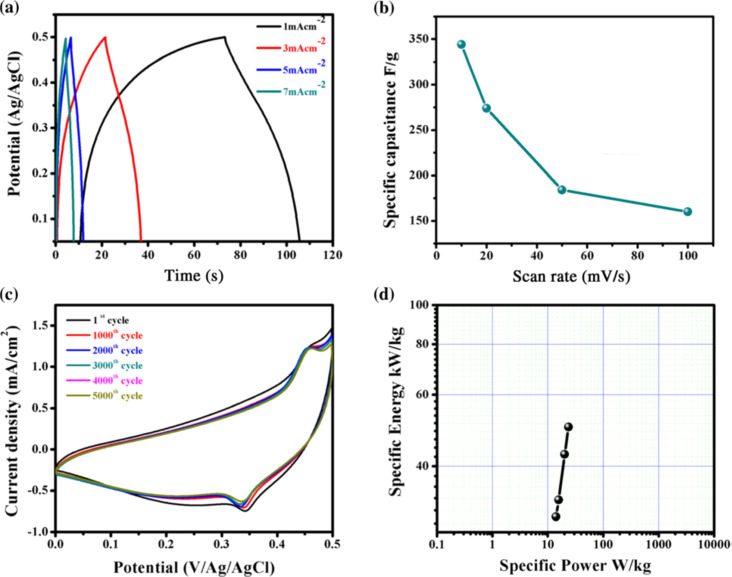
(a) Representation of galvanostatic charge–discharge curves of CuO at diverse current densities. (b) Change of specific capacitance with scan rate (c) study of stability of CuO thin films (d) specific energy *versus* specific power of Ragone plot.^149^

To examine the charge–discharge properties of CuO, the chronoamperometry technique was applied. Fig. 26(a) demonstrates galvanostatic charge–discharge curves at various current densities for CuO and signify a good capacitive behavior of CuO electrode as ref. 152. In Fig. 26(b), the difference of specific capacitance with various scan rates was displayed, which enhanced exponentially with decreasing scan rate.^153^ The electrochemical stability of CuO film electrode was examined by applying CVs at a scan rate of 100 mV s^−1^ for 5000 cycles. Fig. 26(c) demonstrated the cyclic voltammetry scan of CuO film electrode after the 1st to 5000th cycles and confirmed cyclic stability of 83% after 5000 cycles. By using the Ragone plot, the highest values of specific power and specific energy were measured as 3 and 14.1 W h kg^−1^, respectively, using the GCD technique at a current density of 1 mA cm^−2^ for CuO electrode attained in the potential range from 0 to 0.5 V as shown in Fig. 26(d).

Moreover, the electrochemical investigation of Ni-doped CuO nanolayers modified with Ni foam electrodes synthesized by Lobinsky *et al.* revealed the specific capacitance of 154 mA h g^−1^ (1240 F g^−1^) at a current density of 1 A g^−1^,^135^ as already discussed in the doping section. Thus, SILAR-grown CuO material can be potential usage as an electroactive resource for alkaline batteries and pseudo-capacitors.

### 5.5. Photoelectrochemical characterization

**Fig. 27:**
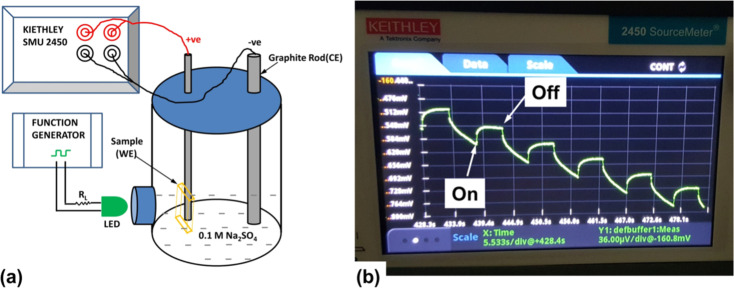
(a) A representation of the transient surface photovoltage measurement system; (b) measurement of a typical Cu_2_O/FTO electrode in a PEC with 0.1 M Na_2_SO_4_ electrolyte followed by real-time measurement in SMU 2450 demonstrating the shape of the LED modulated transient surface photovoltage.^36^

The photo-responsive performance of m-SILAR grown Cu_2_O/FTO electrodes was studied by Farhad and co-workers, through transient surface photovoltage under periodic illumination of a green LED by a HITACHI VG-4429 generator with ∼0.1 Hz–square wave for ‘5 s ON and 5 s OFF’ cycle.^36^ The generated surface photovoltage of the Cu_2_O/FTO electrode was observed by a Keithley SMU 2450 by employing Cu_2_O/FTO thin films as a working electrode, a graphite rod as a counter electrode and 0.1 M Na_2_SO_4_ aqueous solution as an electrolyte as established in Fig. 27(a) and (b). In the presence of an aqueous electrolyte, upon 2500 s LED exposure of the photocathode, the estimated *V*_oc_ for the samples grown with the non-optimized precursor, optimized precursor, and optimized precursor with CH_3_COOH precursor solutions, were observed as 247 ± 38 μV, 36.0 ± 2.0 mV and 47 ± 8 μV respectively. The large *V*_oc_ value projected for optimized precursor film revealed a better Schottky junction produced at Cu_2_O/electrolyte interface, consequently, advocating a better optoelectronic quality of Cu_2_O thin film. The transient surface photovoltage and *V*_oc_ retention for 5000 s, advocating better stability of the SILAR fabricated Cu_2_O thin films in aqueous electrolyte.

## 6. Conclusion

In summary, Cu_*x*_O thin films have been extensively studied and are receiving profound attention because of their fascinating properties and promising uses in a variety of fields. In this article, an inclusive review of the state-of-the-art research activities of diverse Cu_*x*_O thin films was represented based on the SILAR method. This technique has fascinated substantial attention because of its simplicity and low cost, demands less time, and is fit for the large-scale growth of Cu_*x*_O. The morphology, as well as diverse properties of Cu_*x*_O, can be monitored by altering the number of SILAR cycles, the pH of precursor solutions, types of salt, bath temperature, annealing, doping, and the dipping time allowed for reactions. However, the technique does not yet allow for precise control of Cu_*x*_O particle sizes, which can affect the power conversion efficiency in optoelectronic devices. The main limitation of this technique is the high rate of surface roughness as well as less study of the defects in the deposited sample which is very important to control the optical as well as electrical properties in optoelectronics. Having the optimum amount of the deposited Cu_*x*_O is a very significant factor in improving optoelectronic performance. Thus, the inclusion of ligands, complexing agents and surfactants in the precursor solution employed during the SILAR growth could advance the stability of Cu_*x*_O. Precise control of Cu_*x*_O fabrication could accelerate multiple exciton generation effects, leading to a development of overall efficiency.

## Conflicts of interest

There are no conflicts to declare.

## Supplementary Material
